# Supplementary material for: System-wide transcriptome damage and tissue identity loss in COVID-19 patients
Source: Cell Rep Med. 2022 Jan 24;3(2):100522. doi: 10.1016/j.xcrm.2022.100522 (PMC8784611; doi:10.1016/j.xcrm.2022.100522)
Supplement: Document S1. Figures S1–S6 [file mmc1.pdf]

Cell Reports Medicine, Volume *3*

Supplemental information

System-wide transcriptome damage and tissue identity loss in COVID-19 patients

Jiwoon Park, Jonathan Foox, Tyler Hether, David C. Danko, Sarah Warren, Youngmi Kim, Jason Reeves, Daniel J. Butler, Christopher Mozsary, Joel Rosiene, Alon Shaiber, Evan E. Afshin, Matthew MacKay, André F. Rendeiro, Yaron Bram, Vasuretha Chandar, Heather Geiger, Arryn Craney, Priya Velu, Ari M. Melnick, Iman Hajirasouliha, Afshin Beheshti, Deanne Taylor, Amanda Saravia-Butler, Urminder Singh, Eve Syrkin Wurtele, Jonathan Schisler, Samantha Fennessey, André Corvelo, Michael C. Zody, Soren Germer, Steven Salvatore, Shawn Levy, Shixiu Wu, Nicholas P. Tatonetti, Sagi Shapira, Mirella Salvatore, Lars F. Westblade, Melissa Cushing, Hanna Rennert, Alison J. Kriegel, Olivier Elemento, Marcin Imielinski, Charles M. Rice, Alain C. Borczuk, Cem Meydan, Robert E. Schwartz, and Christopher E. Mason

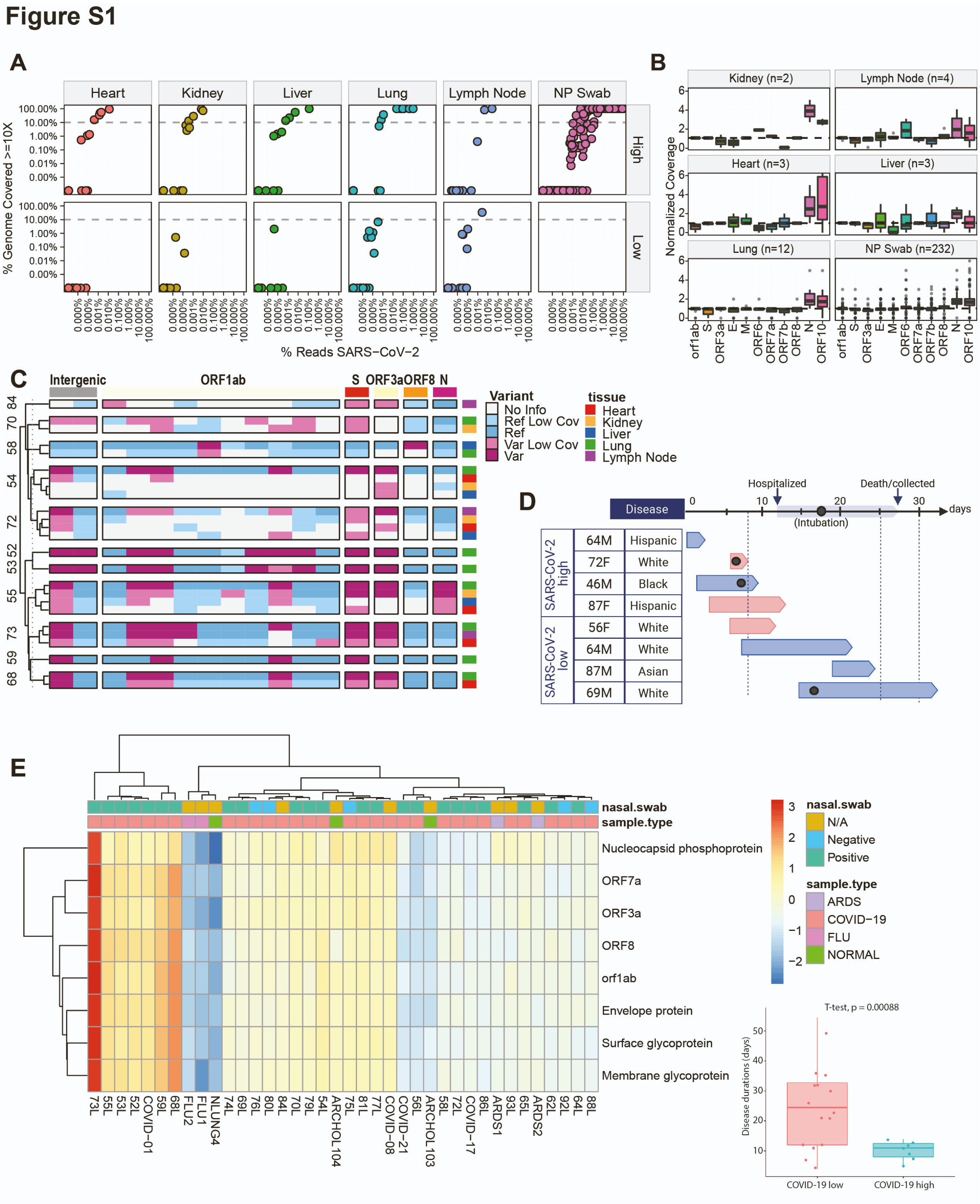

Figure S1, related to Figure 1. **Viral Genome Reconstruction from Total RNA-Seq Data and clinical course of the COVID-19 lung samples.** (**A**) For each patient sample per tissue (41 heart, 27 kidney, 40 liver, 40 lung, 27 lymph node tissues total), the percentage of Total RNA-seq reads mapped uniquely to SARS-CoV-2 and no other taxon is shown, against the percentage of the viral genome (Wuhan reference) that was covered with >10 reads during genome assembly. (**B**) Normalized expression value per gene body in the SARS-CoV-2 genome for samples where at least 10% of the viral genome was covered with >10 reads. (**C**) Heatmap of variant alleles across the viral genome, grouped by patient. (**D**) Summarized clinical course of the COVID-19 patients including four representative samples from high and low viral RNA load, which were used for GeoMx analysis. (**E**) ERCC and HK normalized nCounter gene expression values were centered and rescaled. The resulting Z-scores are shown in this unsupervised clustering. COVID-19 positivity, where applicable, is indicated above

columns. Bottom right included the summary of days of disease durations of each high/low groups (total n = 38, p-value obtained from paired t-test).

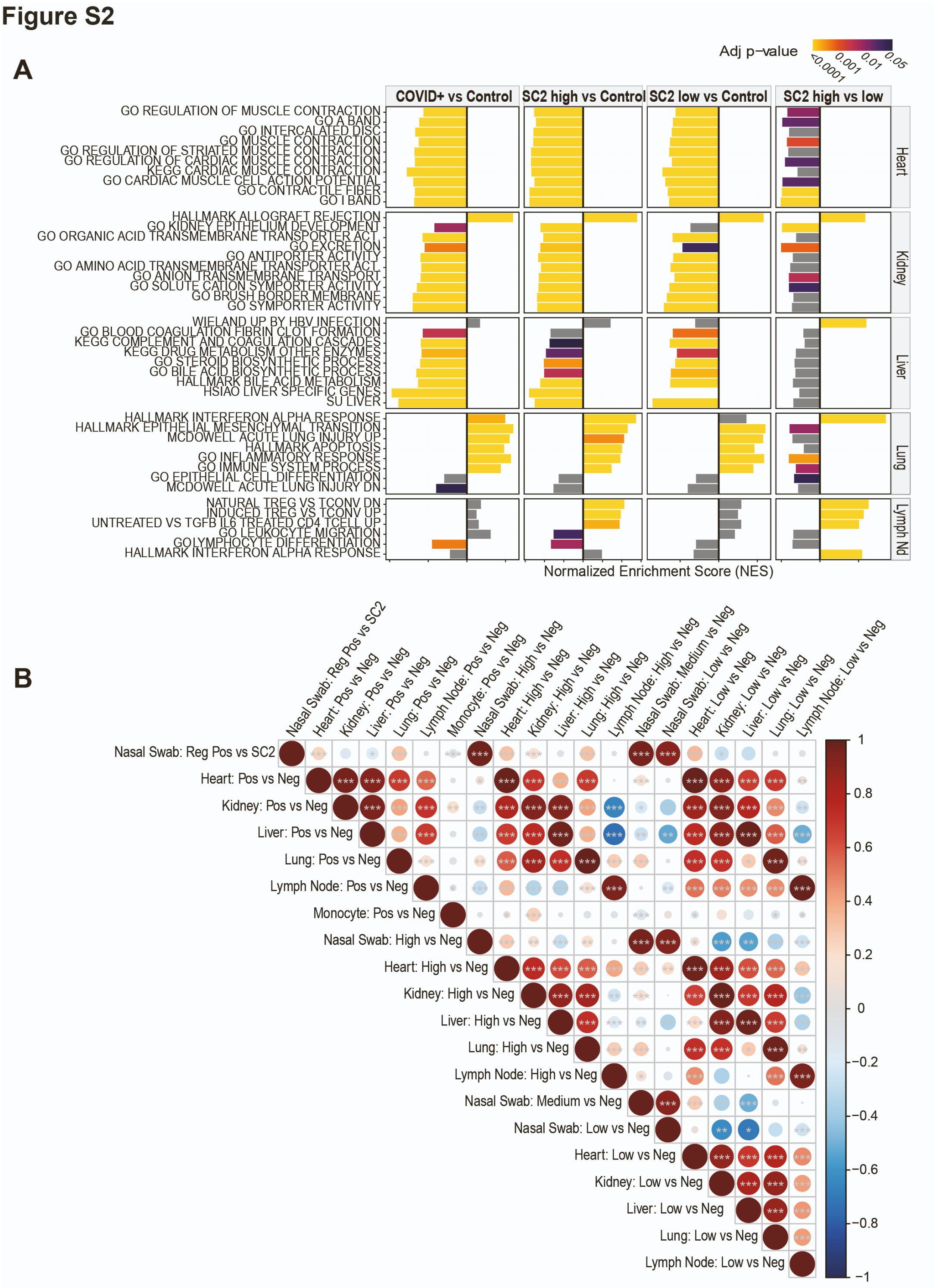

Figure S2, related to Figure 2. **Enriched pathways and correlations observed in COVID-19 by organ types and viral load comparisons**. (A) Select pathways that show significant differences in different tissues (41 heart, 27 kidney, 40 liver, 40 lung, 27 lymph node tissues total) are shown, with the statistical significance shown as a color range (legend, GSEA permutation test and adaptive multilevel splitting Monte Carlo method), and non-significant differences shown in grey. (B) Correlations between each sample type (label) gene expression matrix and other tissue/sample types are shown as a correlation range (red, high up to 1.0 and blue, low down to -1.0). The p-values represent the significance of the correlation with * p-value < 0.05, ** p-value < 0.01, and *** p-value < 0.001.

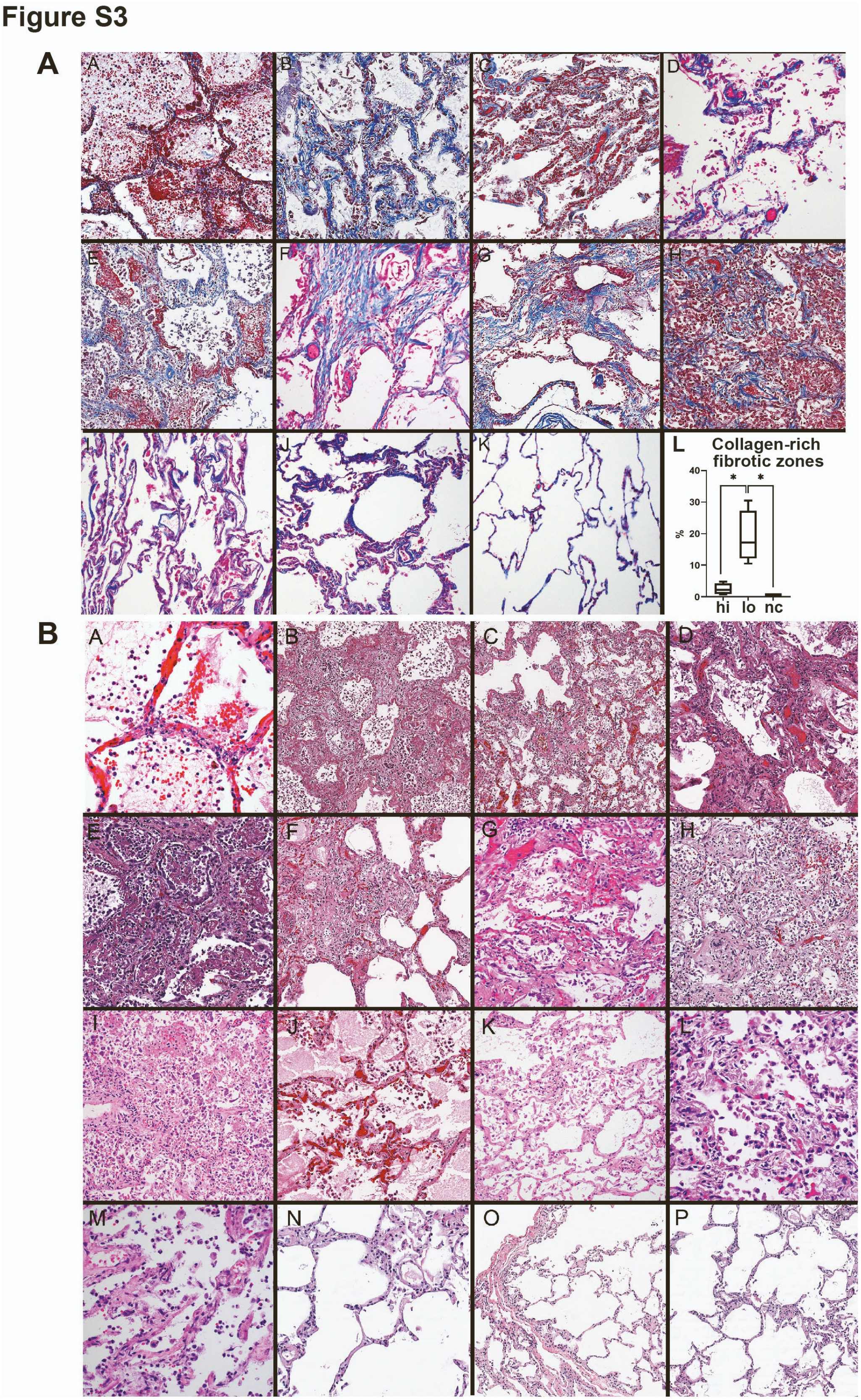

Figure S3, related to Figure 3. **Representative Trichrome stained lung tissue regions used for quantification and GeoMx spatial transcriptomics**. (A) Lung tissue regions for bulk quantification: A-D, COVID-19 with SARS-CoV-2 high cases: COVID1, COVID2, 55 and 59 showing mild increase in collagen zones with focally increased spindled cells, best seen in B in the figure. E-H, COVID-19 with SARS-CoV-2 low cases: COVID 17, 21, 73 and 86 with marked areas of increased

collagen containing spindled cells, best seen in F and G in the figure. I-K, normal lungs: 4, 103 and 104 showing blue staining collagen without expanded zones of cellularity (Masson trichrome, for all panels original magnification x 50). L, Quantification of collagen-rich fibroblastic zones, *:p-value < 0.01. (B) Representative lung morphology for GeoMx spatial profiling: A-H, COVID cases including COVID1, COVID2, 55L, 59L, COVID 17, COVID21, 73L, 86L; I-J, influenza induced ARDS: Flu1, Flu2; K-M, ARDS non-viral: ARDS1, ARDS2, ARDS3; N-P, Normal lung: NL4, Archoi103, Archoi 104. Hematoxylin and eosin stain, Original magnification x100 except for C and D in the figure (x50).

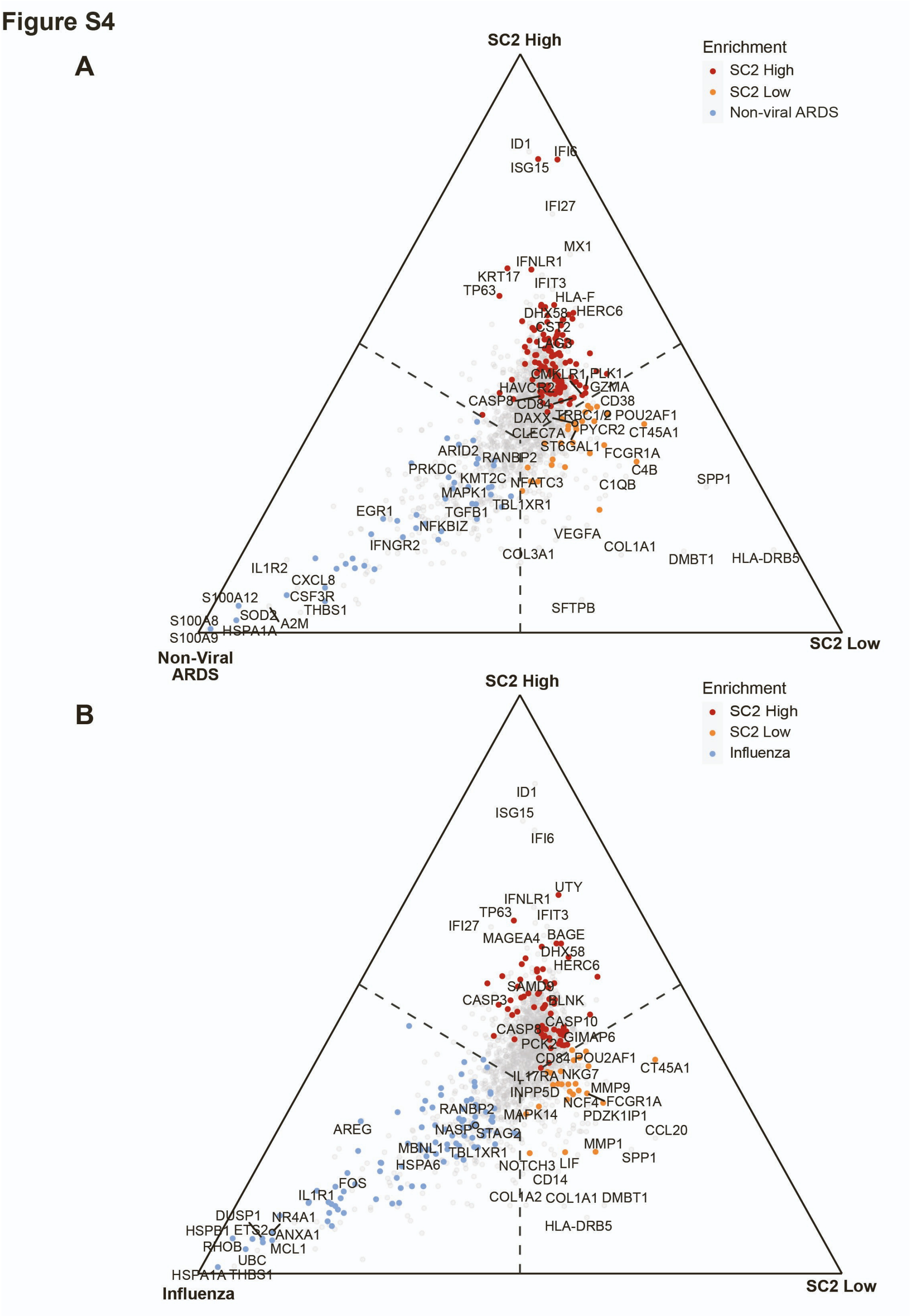

Figure S4, related to Figure 4. **Differentially expressed genes across three conditions**. (**A**) Ternary plot for influenza (n=2, 46 ROIs) vs COVID-19 SARS-CoV-2 high viral load (n=4, 86 ROIs) vs low viral load (n=4, 97 ROIs). (**B**) Ternary plot for non-viral ARDS (n=3, 67 ROIs) vs SARS-CoV-2 high (n=4, 86 ROIs) vs SARS-CoV-2 low (n=4, 97 ROIs).

Figure S5

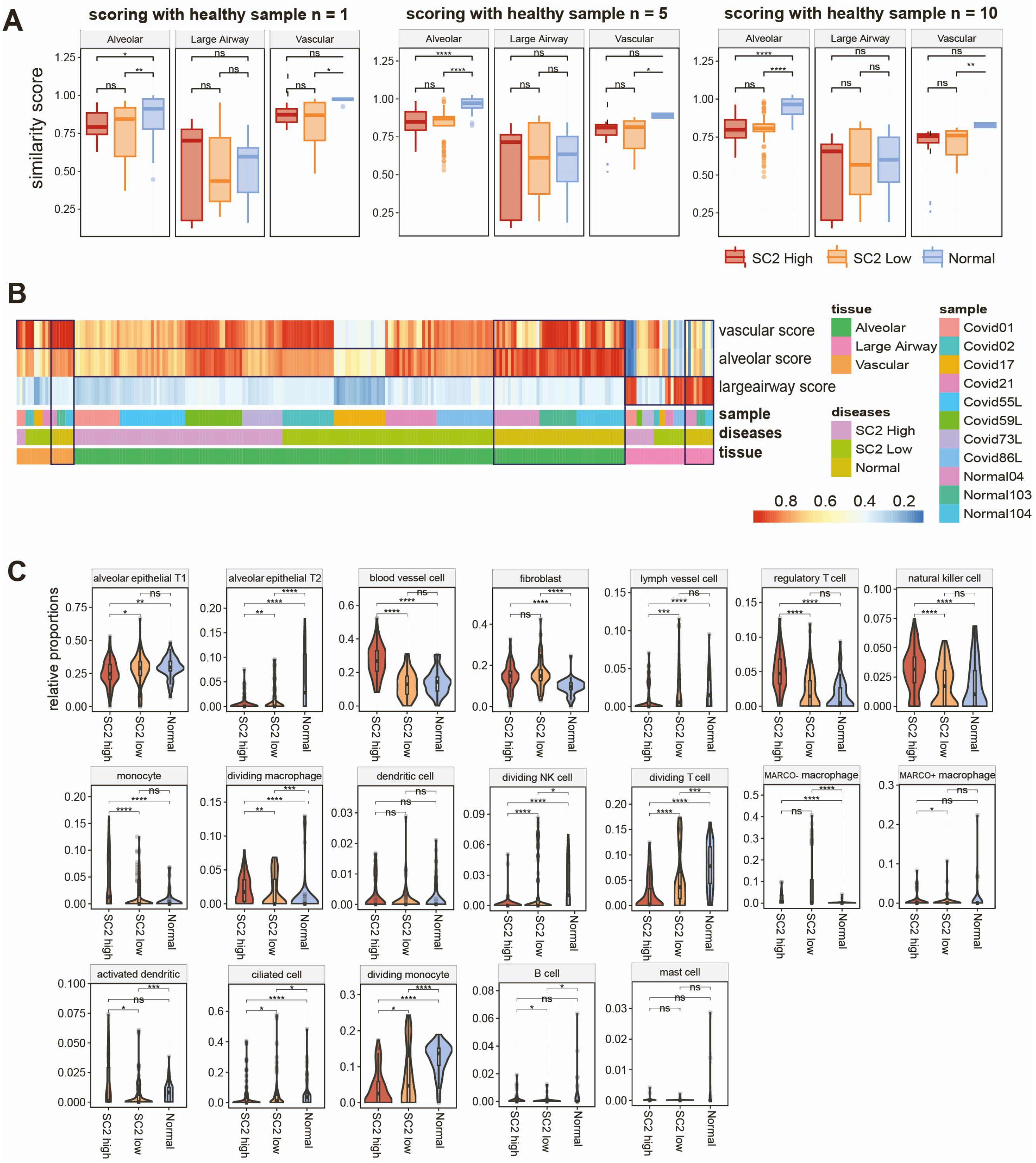

Figure S5, related to Figure 5. **Similarity score** visualizations and cell proportion changes from GeoMx. (**A**) Validation of reference healthy gene profile signature by randomly sampling healthy ROIs (64 ROIs total) for gene expression profiles. (**B**) Heatmap showing individual similarity scores across 64 ROIs. (**C**) Deconvoluted cell type proportions by conditions. From GeoMx data, cell proportions for each ROIs were plotted to compare between COVID-19 (183 ROIs) vs. normal control (64 ROIs). The median and quartiles are noted by the box plot inside. P-value two-tailed t-tests were done to compare the means (ns: non-significant, *:p <= 0.05, **: p <= 0.01, ***: p <= 0.001, and ****: p <= 0.0001).

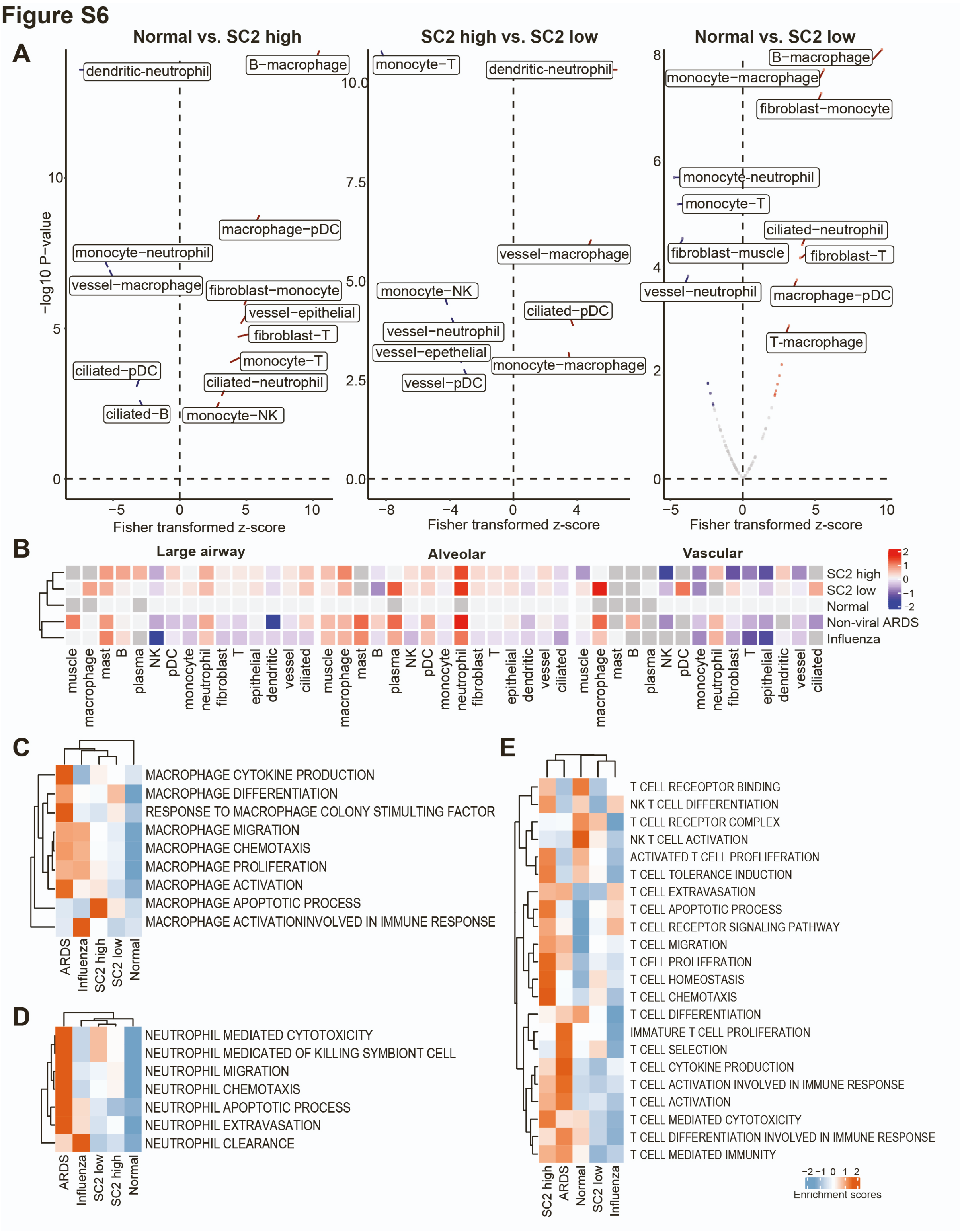

Figure S6, related to Figure 6. **Quantitative descriptions of correlations, cell-type counts entropy values, and ssGSEA scores by conditions**. (**A**) Quantitative, pairwise comparison of the cell type correlations. To quantitatively compare correlation patterns in Figure 6A, the correlations (from 64 normal ROIs, 86 and 97 COVID-19 high and low ROIs) are compared pairwise by Fisher's test. The transformed scores and p-values are shown. (**B**) Entropy Calculations (Maximum likelihood model) for cell-type counts of each condition: SARS-CoV-2 high (n=4, 86 ROIs), low (n=4, 97 ROIs), Influenza (n=2, 46 ROIs), and ARDS (n=3, 67 ROIs), relative to normal (n=3, 64 ROIs). The single-sample enrichment scores of (**C**) macrophage, (**D**) neutrophil, and (**E**) T and NK cell-related gene sets were evaluated. The scores were averaged and compared across SARS-CoV-2 high (n=4, 86 ROIs), low (n=4, 97 ROIs), Influenza (n=2, 46 ROIs), and ARDS (n=3, 67 ROIs), relative to normal (n=3, 64 ROIs).
