# Supplementary material for: System-wide transcriptome damage and tissue identity loss in COVID-19 patients
Source: Cell Rep Med. 2022 Jan 24;3(2):100522. doi: 10.1016/j.xcrm.2022.100522 (PMC8784611; doi:10.1016/j.xcrm.2022.100522)
Supplement: Document S2. Article plus supplemental information [file mmc5.pdf]

Cell Reports
Medicine

Article

System-wide transcriptome damage and tissue identity loss in COVID-19 patients

Graphical abstract

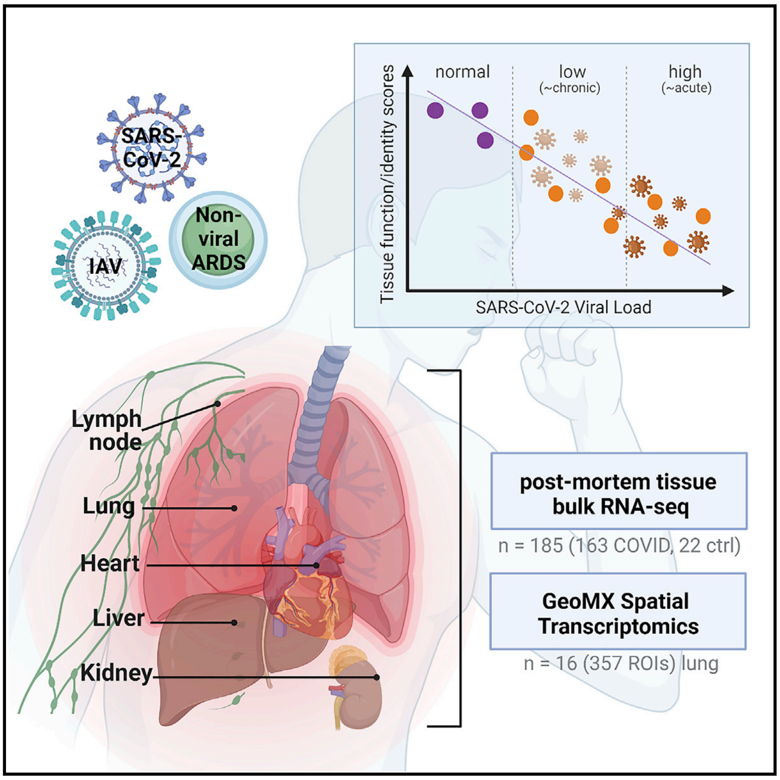

Authors

Jiwoon Park, Jonathan Foox, Tyler Hether, ..., Cem Meydan, Robert E. Schwartz, Christopher E. Mason

Correspondence

alb9003@med.cornell.edu (A.C.B.),
cem2009@med.cornell.edu (C.M.),
res2025@med.cornell.edu (R.E.S.),
chm2042@med.cornell.edu (C.E.M.)

In brief

Park et al. report system-wide transcriptome damage and tissue identity loss wrought by SARS-CoV-2, influenza, and bacterial infection across multiple organs (heart, liver, lung, kidney, and lymph nodes) and provide a spatiotemporal landscape of COVID-19 in the lung.

Highlights

- Across all organs, fibroblast, and immune cell populations increase in COVID-19 patients
- Organ-specific cell types and functional markers are lost in all COVID-19 tissue types
- Lung compartment identity loss correlates with SARS-CoV-2 viral loads
- COVID-19 uniquely disrupts co-occurrence cell type clusters (different from IAV/ARDS)

Park et al., 2022, Cell Reports Medicine *3*, 100522
February 15, 2022 © 2022 The Author(s).
https://doi.org/10.1016/j.xcrm.2022.100522

CellPress

Cell Reports Medicine

CellPress
OPEN ACCESS

Article

System-wide transcriptome damage and tissue identity loss in COVID-19 patients

Jiwoon Park,[1,2] Jonathan Foox,[1,3] Tyler Hether,[4] David C. Danko,[1,3,5] Sarah Warren,[4] Youngmi Kim,[4] Jason Reeves,[4] Daniel J. Butler,[1] Christopher Mozsary,[1] Joel Rosiene,[6,7] Alon Shaiber,[6,7] Evan E. Afshin,[1,3] Matthew MacKay,[1] André F. Rendeiro,[3,9] Yaron Bram,[8] Vasuretha Chandar,[8] Heather Geiger,[6] Arryn Craney,[7] Priya Velu,[7] Ari M. Melnick,[8] Iman Hajirasouliha,[1,3,9] Afshin Beheshti,[10,11] Deanne Taylor,[12,13] Amanda Saravia-Butler,[14,15] Urminder Singh,[16] Eve Syrkin Wurtele,[16] Jonathan Schisler,[17,18] Samantha Fennessey,[6] André Corvelo,[6] Michael C. Zody,[6] Soren Germer,[6]

(Author list continued on next page)

[1]Department of Physiology, Biophysics and Systems Biology, Weill Cornell Medicine, New York, NY, USA
[2]Laboratory of Virology and Infectious Disease, The Rockefeller University, New York, NY 10065, USA
[3]The HRH Prince Alwaleed Bin Talal Bin Abdulaziz Alsaud Institute for Computational Biomedicine, Weill Cornell Medicine, New York, NY, USA
[4]NanoString Technologies, Inc., Seattle, WA, USA
[5]Tri-Institutional Computational Biology & Medicine Program, Weill Cornell Medicine, New York, NY, USA
[6]New York Genome Center, New York, NY, USA
[7]Department of Pathology and Laboratory Medicine, Weill Cornell Medicine, New York, NY, USA
[8]Department of Medicine, Weill Cornell Medicine, New York, NY, USA
[9]Englander Institute for Precision Medicine and the Meyer Cancer Center, Weill Cornell Medicine, New York, NY, USA
[10]KBR, Space Biosciences Division, NASA Ames Research Center, Moffett Field, CA, USA
[11]Stanley Center for Psychiatric Research, Broad Institute of MIT and Harvard, Cambridge, MA, USA
[12]Department of Biomedical and Health Informatics, The Children's Hospital of Philadelphia, Philadelphia, PA, USA
[13]Department of Pediatrics, Perelman School of Medicine, University of Pennsylvania, Philadelphia, PA, USA
[14]Space Biosciences Division, NASA Ames Research Center, Moffett Field, CA, USA
[15]Logyx, LLC, Mountain View, CA, USA
[16]Bioinformatics and Computational Biology Program, Center for Metabolic Biology, Department of Genetics, Development and Cell Biology Iowa State University, Ames, IA, USA
[17]McAllister Heart Institute at The University of North Carolina at Chapel Hill, Chapel Hill, NC, USA

(Affiliations continued on next page)

SUMMARY

The molecular mechanisms underlying the clinical manifestations of coronavirus disease 2019 (COVID-19), and what distinguishes them from common seasonal influenza virus and other lung injury states such as acute respiratory distress syndrome, remain poorly understood. To address these challenges, we combine transcriptional profiling of 646 clinical nasopharyngeal swabs and 39 patient autopsy tissues to define body-wide transcriptome changes in response to COVID-19. We then match these data with spatial protein and expression profiling across 357 tissue sections from 16 representative patient lung samples and identify tissue-compartment-specific damage wrought by severe acute respiratory syndrome coronavirus 2 (SARS-CoV-2) infection, evident as a function of varying viral loads during the clinical course of infection and tissue-type-specific expression states. Overall, our findings reveal a systemic disruption of canonical cellular and transcriptional pathways across all tissues, which can inform subsequent studies to combat the mortality of COVID-19 and to better understand the molecular dynamics of lethal SARS-CoV-2 and other respiratory infections.

INTRODUCTION

In March 2020, the World Health Organization (WHO) declared a novel pandemic of coronavirus disease 2019 (COVID-19), an infection caused by the betacoronavirus severe acute respiratory syndrome coronavirus 2 (SARS-CoV-2), which is currently attributed to over 320 million cases and over 5.5 million deaths globally (https://coronavirus.jhu.edu).[1] Since the presenting

This is an open access article under the CC BY license (http://creativecommons.org/licenses/by/4.0/).

Steven Salvatore,[7] Shawn Levy,[19] Shixiu Wu,[20,21] Nicholas P. Tatonetti,[22] Sagi Shapira,[22] Mirella Salvatore,[8,23] Lars F. Westblade,[7,8] Melissa Cushing,[7] Hanna Rennert,[7] Alison J. Kriegel,[24] Olivier Elemento,[1,5,9] Marcin Imielinski,[6,7] Charles M. Rice,[2] Alain C. Borczuk,[7,*] Cem Meydan,[1,3,*] Robert E. Schwartz,[1,8,*] and Christopher E. Mason[1,3,6,25,26,*]

[18]Department of Pharmacology, and Department of Pathology and Lab Medicine at The University of North Carolina at Chapel Hill, Chapel Hill, NC, USA
[19]HudsonAlpha Discovery Institute, Huntsville, AL, USA
[20]Hangzhou Cancer Institute, Hangzhou Cancer Hospital, Hangzhou, China
[21]Department of Radiation Oncology, Hangzhou Cancer Hospital, Hangzhou, China
[22]Department of Biomedical Informatics, Department of Systems Biology, Department of Medicine, Institute for Genomic Medicine, Columbia University, New York, NY, USA
[23]Department of Population Health Sciences, Weill Cornell Medicine, New York, NY, USA
[24]Department of Physiology, Cardiovascular Center, Center of Systems Molecular Medicine, Medical College of Wisconsin, Milwaukee, WI, USA
[25]The Feil Family Brain and Mind Research Institute, Weill Cornell Medicine, New York, NY, USA
[26]Lead contact
*Correspondence: alb9003@med.cornell.edu (A.C.B.), cem2009@med.cornell.edu (C.M.), res2025@med.cornell.edu (R.E.S.), chm2042@med.cornell.edu (C.E.M.)
https://doi.org/10.1016/j.xcrm.2022.100522

symptoms of COVID-19 resemble those of common viral respiratory infections, a molecular diagnosis is required to distinguish a SARS-CoV-2 infection from influenza and other respiratory illnesses,[2,3] and ongoing questions remain about the host responses to SARS-CoV-2 relative to other respiratory pathogens. As severe illness and death continue to impact a segment of COVID-19-positive individuals, urgent questions persist about the molecular drivers of morbidity and mortality associated with SARS-CoV-2 infection. This knowledge could lead to improvements in both the acute treatment and the long-term management of pathological changes in multiple organs.

Prior work has shown that COVID-19 leads to high systemic interferon responses (both alpha and gamma) and that co-infection with other pathogens is relatively rare (3%–15%).[4–6] Yet there are limited data for discriminating the molecular response between different kinds of respiratory infections or pulmonary conditions (e.g., influenza A (IAV) versus SARS-CoV-2 infections) and almost no data on the variegated impact of different pathogens across different human tissues other than clinical observations from intensive care unit (ICU) patients.[7] Delineation of pathogen- and tissue-specific differences is critical for understanding the molecular determinants of mortality associated with COVID-19 and for developing novel diagnostics and therapeutic interventions.

To address this knowledge gap, we used shotgun metatranscriptomics (total RNA sequencing [RNA-seq]) to comprehensively profile human tissues in 39 patients who died from COVID-19 (185 total autopsy samples), including heart, liver, lung, kidney, and lymph nodes, analyzed gene expression, and assessed the system-wide impact of SARS-CoV-2 infection. We also used a spatial protein and transcript mapping platform (GeoMx) to visualize the cartography of the infection in these tissues and to discover disruption of regional and cell-type-specific expression. The spatial transcriptomics data examined 357 total regions of interest (ROIs), which were selected from 13 patients who had SARS-CoV-2, influenza, or bacterial infections and from 3 normal patients as controls, revealing the cellular and regulatory signatures that define these distinct pathological states. Finally, to provide context to earlier stages and sites of infection, we compared these in-depth spatial and tissue-specific transcriptome maps with an independent cohort of nasopharyngeal (NP) swabs from 216 COVID-19-positive patients and 430 COVID-19-negative controls, which revealed a significant and distinct disruption of cellular and transcriptional programs induced by SARS-CoV-2 infection in the patients who unfortunately succumbed to the disease. As a resource for the field, these data have also been placed in an online portal (https://covidgenes.weill.cornell.edu/) for additional data mining and visualization.

RESULTS

System-wide host responses and transcriptome changes by COVID-19

We first used shotgun metatranscriptomics (total RNA-seq) for host and viral profiling on 39 patients who died from COVID-19, including 185 organ-specific tissue samples from the respective autopsies and 22 healthy control samples from organ donor remnant tissues (Figure 1A; Table S1). We examined the COVID-19-specific host responses and transcriptome changes across various organs (heart, kidney, liver, lung, and lymph nodes) to ascertain the differentially expressed genes (DEGs) between COVID-19 and control sample sets ($q < 0.01$, expression fold change > 1.5 by the differential expression analysis method [DESeq2]; Figure 1B; Table S2). Separately, SARS-CoV-2 RNA reads were aligned to the SARS-CoV-2 genome and the number of reads was discerned across multiple tissues including the lung, lymph node, kidney, liver, and heart (Figure S1) but mostly in the lung. Viral reads were robustly detected in NP swab samples from COVID-19 patients, consistent with previous reports.[4,8] Normalized coverage values in SARS-CoV-2-positive autopsy tissue samples revealed a detection bias toward the SARS-CoV-2 3′ end sequences, which is consistent with the known viral transcript abundance (Figures S1A and S1B).[9] Reconstruction of the viral genomes revealed known and unknown variants common to many patients and some evidence of intra-host variability (Figure S1C). Coverage limitations notwithstanding, we generally saw evidence of the same viral strain across the organ systems within each patient. We also assessed the variability of the COVID-19 patient samples by clustering based on the SARS-CoV-2 viral loads (and noted as SC2

CellPress
OPEN ACCESS

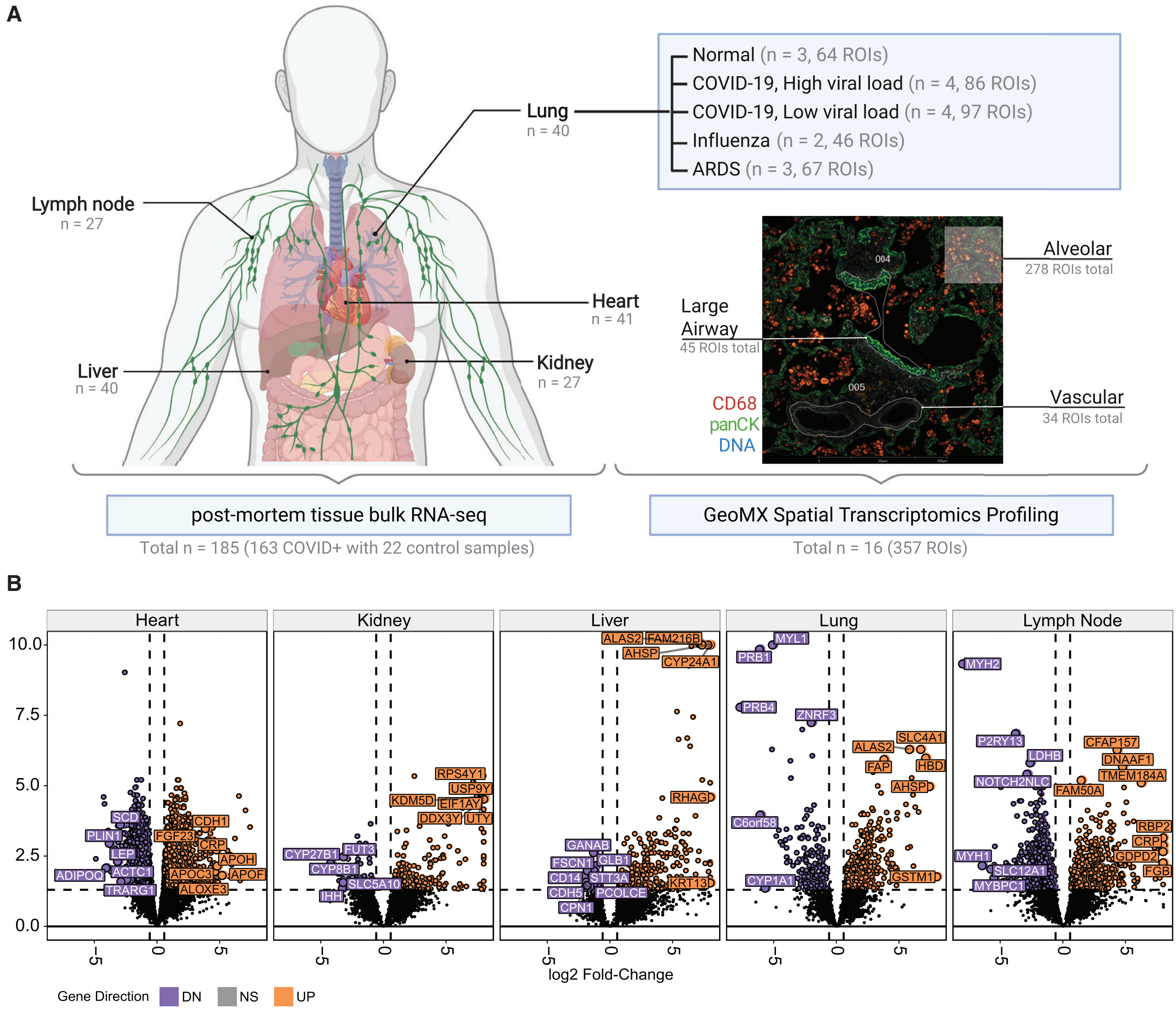

Figure 1. Tissue- and duration-specific dysregulation of gene expression from SARS-CoV-2 infection

(A) Sample overview by tissue for RNA-seq and GeoMx experiments. Number of regions of interest (ROIs) and patient numbers (n) are summarized, and a representative tissue slide image for GeoMX spatial profiling is presented along with the tissue types.

(B) Volcano plots of the COVID-19-positive versus normal tissues are shown for the five tissues from autopsy: heart (n = 41), kidney (n = 27), liver (n = 40), lung (n = 40), and lymph node (n = 27). Differentially expressed genes (>1.5-fold, q < 0.05, DESeq2) are shown as purple spots (downregulated in COVID-19) and orange (upregulated in COVID-19).

high and SC2 low), and the viral loads were inversely correlated with the duration of disease and independent of factors such as race, age, or gender (Figures S1D and S1E). In addition to the annotated clinical metadata table (Table S1), we summarized the clinical courses of a few representative COVID-19 patients from hospitalization to intubation to death (Figure S1D).

COVID-19 pathway enrichment analysis revealed significant changes (q < 0.01) in pathways for viral infection (regulation of viral genome replication and viral entry into host cell) and immune response (regulation of type 1 interferon response and regulation of tyrosine phosphorylation of Stat protein, Gene Ontology (GO) Regulation of toll-like receptor signaling pathway), and the enrichment varied as a function of the viral load (Figure 2A; Table S2). We observed that each tissue showed its own distinct transcriptional disruption in response to SARS-CoV-2 infection, with the lymph node exhibiting the greatest number of DEGs when compared with controls (in both high- and low-viral-load groups). Of note, both tissue-specific and pan-tissue disruptions of normal-expression programs were observed (Figure 1B), and these were then summarized using gene set enrichment analysis (GSEA; Figures 2A and S2A). Some pathways were consistently dysregulated in all tissues during early infection (SARS-CoV-2 high), such as the G2M checkpoint (q values of 6.4×10^{-19}, 3.0×10^{-5}, $1.2 \times$

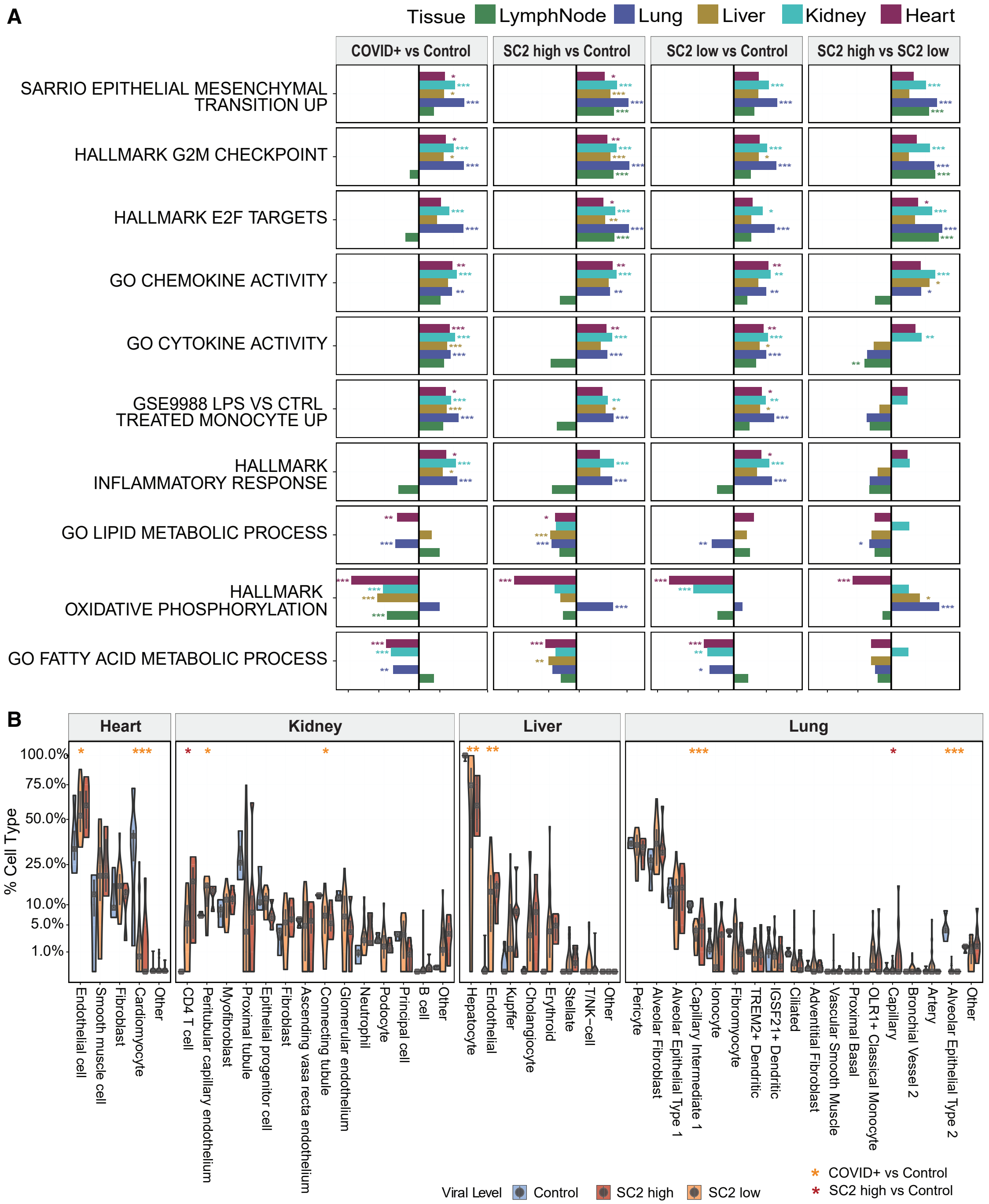

Figure 2. Pathways and cell population changes of COVID-19

(A) The pathways that show significant differences in all or the majority of the five tissue types are shown, with statistical significance from GSEA testing across five kinds of tissue (colors in legend), including heart (n = 41), kidney (n = 27), liver (n = 40), lung (n = 40), and lymph node (n = 27). The x axis shows the normalized enrichment score for COVID-19-positive versus control, SARS-CoV-2 high or low versus control, and SARS-CoV-2 high versus Low comparisons.

(B) Cellular deconvolution distribution violin plots for the heart, kidney, liver, and lung. SARS-CoV-2 high (red), low (orange), and normal (blue) tissues are shown using a square root scale. The number of biological replicates is the same as in (A).

10^{-8}, 9.7×10^{-8}, and 0.002 for lung, liver, kidney, lymph node, and heart, respectively; Table S2), E2F targeting (q values of 2.8×10^{-26}, 0.00672, 1.9×10^{-7}, 9.9×10^{-9}, and 0.047, respectively), and epithelial mesenchymal transition (EMT; q values of 2.1×10^{-22}, 3.0×10^{-4}, 2.9×10^{-7}, 1.4×10^{-7}, and 0.0287, respectively), but in the late infection

(SARS-CoV-2 low), gene networks showed more inter-tissue heterogeneity in their disrupted pathways, including cytokine activity and inflammatory response. However, in both the SARS-CoV-2 high- and low-viral-load groups, the G2M checkpoint and E2F networks were consistently upregulated, indicating a core, persistent set of dysregulated cell-cycle regulation genes during early and late stages of infection.

The DEGs and GSEA results were then examined for the largest differences between the infection level and stage (SARS-CoV-2 high, early COVID-19 infection versus SARS-CoV-2 low, late COVID-19 infection). Interestingly, the heart tissues showed the largest transcriptional differences, revealing that the later stage of the infection had a much greater impact on cardiac tissues (Figure S2A). To place these results into a larger context and to compare them with the findings of other data sets, we compared DEGs from each tissue with RNA-seq data from NP swabs as well as RNA-seq data from a publicly available data set on monocytes from COVID-19-positive and -negative patients (Figure S2B).[4] While the highest correlations were seen within the same tissue types, most of the tissues with a high viral load showed a statistically significant, positive correlation with the DEGs in the NP swab samples ($q < 0.01$) when compared with normal/negative patients. In contrast, the later infection (low-viral-load) patients' tissues showed a negative correlation with the NP swab samples, indicating that the systemic impact of SARS-CoV-2 can be missed when not considering the biological impact on different organs. Interestingly, when matched with disease severity (high-, medium-, and low-viral-load groups within NP swab samples), the difference was bigger in the low and medium groups than in the high groups.

To create a more fine-grained analysis of the cellular gene expression states in each tissue, we used the cell deconvolution multiple signal classification algorithm (MuSiC) on each tissue's RNA-seq data (see STAR Methods). The MuSiC results showed distinct disruptions of the transcriptional programs for each type of tissue in the COVID-19 patients and in the gain or loss of cell types (Figure 2B). Consistent with previous reports, the lung showed a loss of the capillary intermediate cells and alveolar epithelial cell types.[10,11] Strikingly, we also found decreases in the major cell types in each organ type, suggesting a systemic disruption of the COVID-19 response. The kidney and liver showed a loss of proximal tubule in the kidney and of hepatocyte marker expressions in the liver but an increase in T cells in both organs. Furthermore, the heart showed a near-complete loss of the cell signatures for cardiomyocytes in both SARS-CoV-2 high and low viral loads (Figures 2B and 3A), despite no obvious gross or histologic changes in the heart (Figure 3B). This observation extends from previously reported cardiovascular involvements in COVID-19 and further defines the SARS-CoV-2-specific transcript and cellular changes in lung as well as other organs such as heart, liver, and kidney.[12,13]

We then looked at several markers related to the functions and processes of each organ (Figure 3C). While the lung showed the biggest changes in response to COVID-19, the loss of functional markers was specific to organ type. For example, surfactant proteins (i.e., SFTPA1, SFTPB, SFTPC), which are components of the alveolar lipoprotein complex crucial for gas exchange, were lost in the lung but increased in other organs. Similarly, markers associated with liver function (i.e., liver enzymes and proteins such as ALB, HAO1, and ALDOB) and solute carrier family proteins (i.e., SLC22A13, SLC34A1, uromodulin [UMOD]) were specifically lost in liver and kidney, respectively. In addition to the cardiomyocyte markers (Figure 3A), we also found that markers such as phospholamban (PLN, calcium pump inhibitor), heart and neural crest derivatives expressed 1 (HAND1), and troponin cardiac type (TNNT2) were specifically lost in the heart. This observation suggests that in addition to cell type losses, the disruptions due to COVID-19 affect the biological processes and functions for which each organ system is responsible. Consistent with recent reports related to virus-induced senescence found in COVID-19 lung models,[14] these data also show that each organ system (lung, heart, liver, and kidney) exhibits its own distinct inflammatory response and resultant change of tissue identity.

Spatial and expression profiling of high and low SARS-CoV-2 infection in the lung

For a deeper examination of COVID-19 lung tissue, we then used the GeoMx Digital Spatial Profiling (DSP) platform to perform multiplexed high-resolution spatial transcriptomic profiling of 357 lung tissue ROIs from 16 patients. The ROIs were selected from deceased patients with COVID-19 (n = 8), nonviral acute respiratory distress syndrome (ARDS) (n = 2), influenza-induced ARDS (n = 3), and healthy tissues from individuals without infections as controls (n = 3) using nCounter Multiplex Analysis incorporated with targets for SARS-CoV-2 (i.e., IO360 panel plus COVID-19 Spike-in; Figure 1A). Among COVID-19 patients characterized with total RNA-seq, we identified patients that had high overall SARS-CoV-2 expression (SC2 high) or had low overall expression of SARS-CoV-2 (SC2 low) from their lung-tissue samples, and four representative samples from each group were selected for further downstream analysis. Serial sections were stained with an RNA scope probe against the viral Spike (S) gene, Syto13 (nuclear DNA), macrophages (CD68), immune cells (CD45), and epithelial cells (pan-cytokeratin) along with the GeoMx Cancer Transcriptome Atlas Panel (CTA, 1,811 targets) supplemented with 23 human genes associated with lung biology and two open reading frames (ORFs) from the SARS-CoV-2 genome (Figures 1A and S1). We chose tissue ROIs that captured three structural components of the lung, including vascular, airway, and alveolar regions (Figure S3).

We observed significant differences across tissue types within the lung (DEGs with $q < 0.05$ and > 1 fold change by DESeq2). For example, vascular ROIs showed an increase in ACTA2 and FLNA, while alveolar regions exhibited a mixture of signals from macrophage and monocyte genes (CD163 and CD68) with collagen (COL1A1, COL1A2, COL6A3). In large airway tissues, genes related to the mucosal layer (MUC4, MUC5AC, MUC5B) as well as cytokine-mediated signaling pathway genes (CCL20, CXCL1, CXCL6, CXCL6, MMP1) and type I interferon genes (IFIT3, ISG15, STAT1, MX1) were upregulated in COVID-19. Regardless of the tissue type, we saw consistent increases in genes such as B2M, CD81, GNAS, HLA.B, HSPA1A, HSPB1, and SERPING1 in COVID-19 lungs (Figure 4A; Table S3). When we summarized these genes using GO terms, tissue-type-specific processes in response to COVID-19 were distinct from overall inflammation (Table S3). While inflammatory

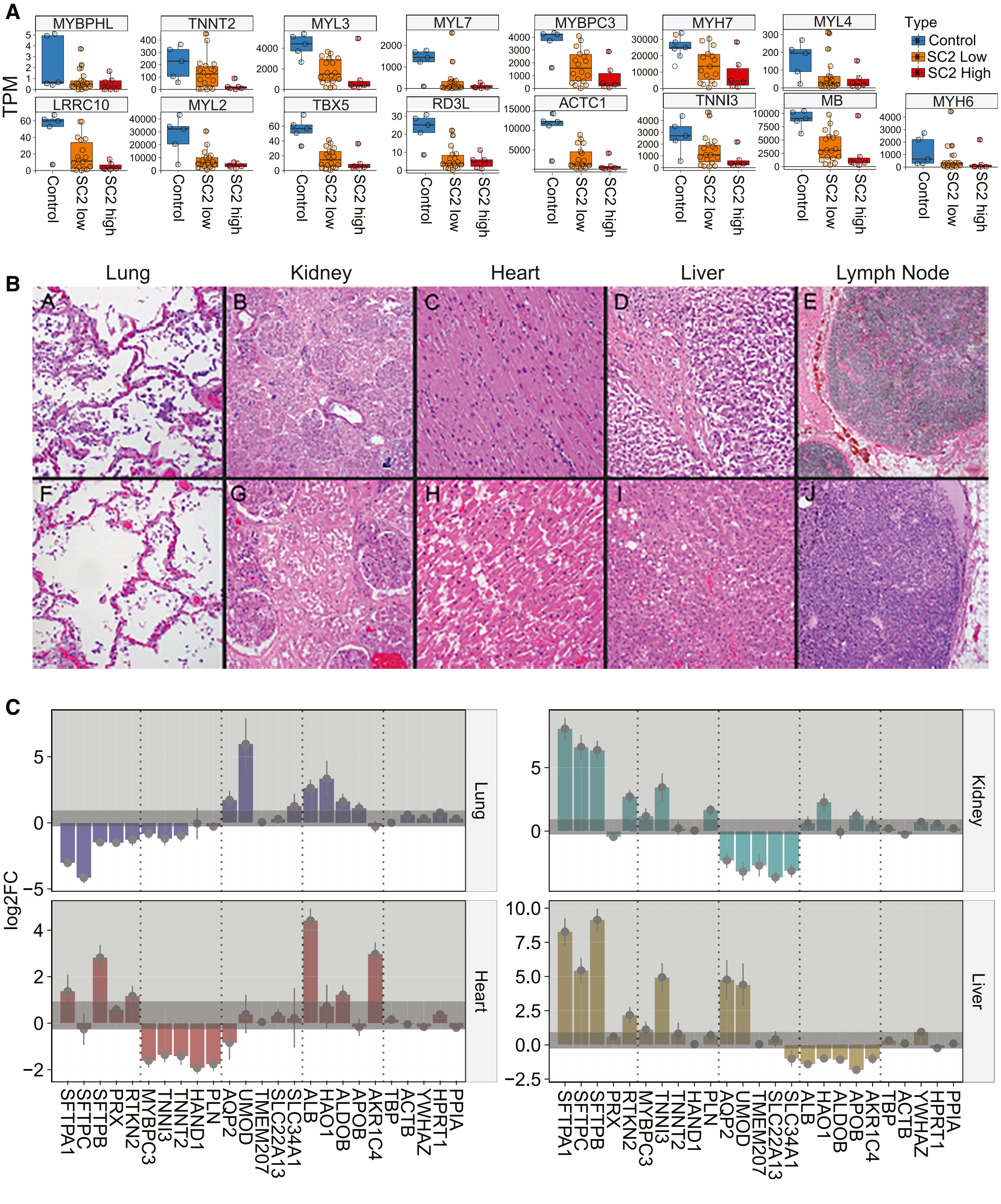

Figure 3. Cellular disruption and tissue identity loss from SARS-CoV-2 infection

(A) Specific gene expression distributions for cardiomyocyte-related genes (from patient autopsy samples, n = 41).

(B) Sample collection strategy for COVID-19 autopsy samples from complete adult cases.

(A–J) Two cases with representative hematoxylin and eosin (H&E) images are shown: (A–E) Cases 58 and (F–J) 73. (A and F) Lung, (B and G) kidney, (C and H) heart, (D and I) liver, and (E and J) mediastinal lymph node (H&E stain). (B, D, E, I, and J) Original magnification ×50. (A, C, F, G, and H) Original magnification ×100.

(C) Gene expression changes of representative functional markers for each organ: heart (n = 41), kidney (n = 27), liver (n = 40), and lung (n = 40). Five genes related to the organ function were chosen (from the Human Protein Atlas), along with five housekeeping genes on the rightmost side. Darker gray shaded area represents the ranges of the housekeeping gene changes (baseline noise) and error bars show standard error from DESeq2 output, relative to the uninfected control.

responses were present in all tissue compartments, macrophage and monocyte activation were prominent in alveolar regions, while fibrosis occurred near the vascular region. Moreover, type I interferon- and cytokine-related pathways were found significant in large airway tissues, while complement activation was found in vascular tissues.

CellPress
OPEN ACCESS

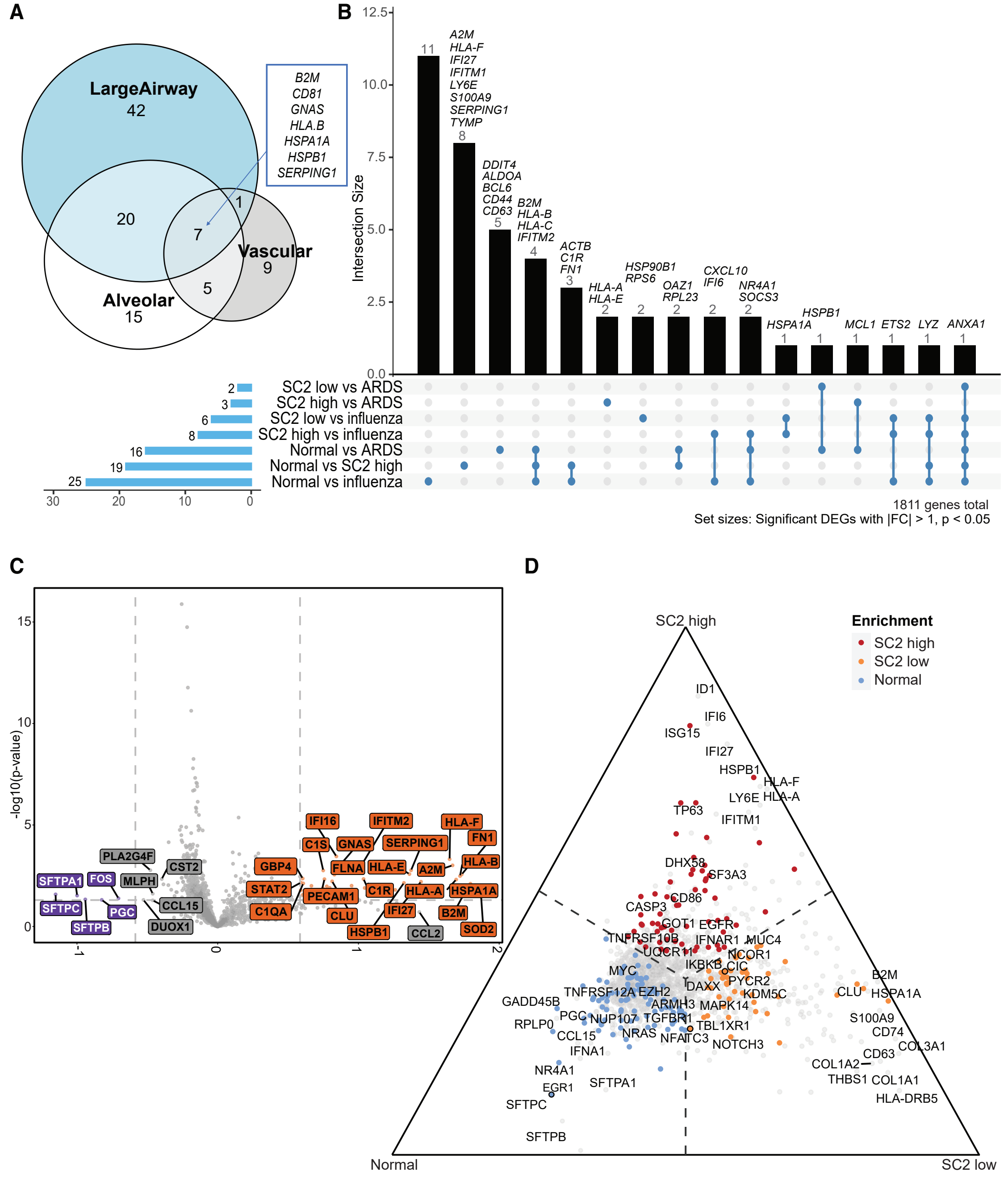

Figure 4. Spatial transcriptomics identifies tissue- and disease-specific differences

(A) Venn diagram of tissue-specific COVID-19 DEGs (relative to normal, adjusted p values < 0.01, >1-fold) from 16 patients across 357 ROIs in total.

(B) UpSet plot depicting intersections of disease-specific DEGs (p values < 0.05, fold change [|FC|] > 1), also from 16 patients across 357 ROIs in total.

(C) Volcano plot showing differences between normal (n = 3, 64 ROIs) and SARS-CoV-2 high-viral-load samples (n = 4, 86 ROIs) after accounting for compartmental differences. Top genes, in terms of p value or FC are indicated in gray, and COVID-19 Spike-in genes are labeled in black.

(D) Ternary plot of a combined analysis of SARS-CoV-2 high (n = 4, 86 ROIs), low (n = 4, 97 ROIs), and normal (n = 3, 64 ROIs), where genes are projected away from the center based on their marginal means. Genes upregulated in a single group approach that group's corner. Top genes in terms of p value or FC are labeled. Genes with color were significant with p < 0.05. Genes with outlines are significant after correcting for multiple hypothesis testing.

We also compared differences across injury sources (SARS-CoV-2 infection, influenza, and nonviral ARDS) and found significant differences between normal and SARS-CoV-2-high samples, even after accounting for compartmental variability (Figures 4B and S4). These differences included a decrease in the expression of surfactant genes (SFTPA1, SFTPB, and

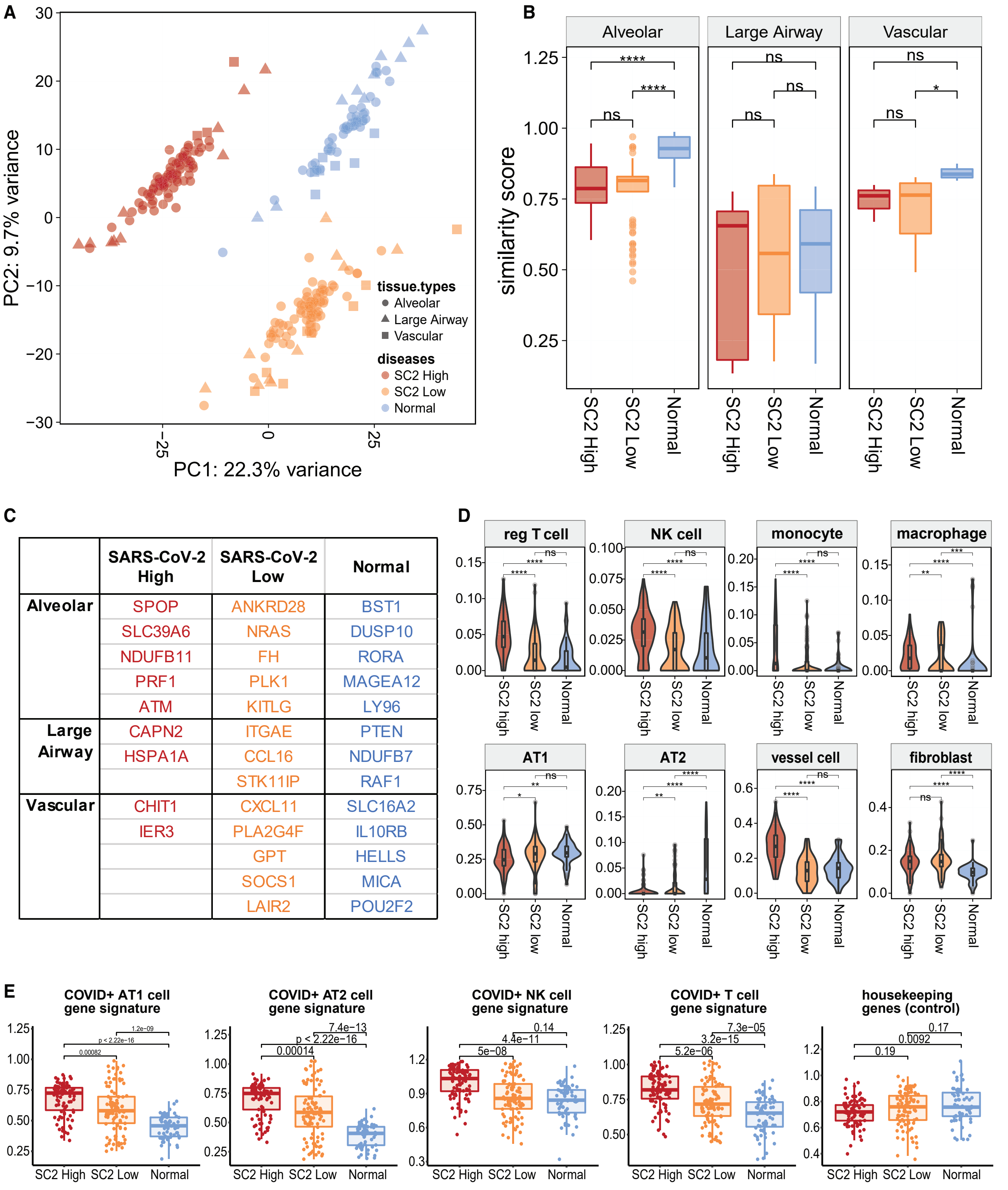

	SARS-CoV-2 High	SARS-CoV-2 Low	Normal
Alveolar	SPOP	ANKRD28	BST1
	SLC39A6	NRAS	DUSP10
	NDUFB11	FH	RORA
	PRF1	PLK1	MAGEA12
	ATM	KITLG	LY96
Large Airway	CAPN2	ITGAE	PTEN
	HSPA1A	CCL16	NDUFB7
		STK11IP	RAF1
Vascular	CHIT1	CXCL11	SLC16A2
	IER3	PLA2G4F	IL10RB
		GPT	HELLS
		SOCS1	MICA
		LAIR2	POU2F2

Figure 5. Evaluation of tissue identity loss and heterogeneity from infection

(A) Principal-component analysis (PCA) to see sample- and disease-clustering. Colors denote disease conditions (64 normal ROIs, 86 and 97 SARS-CoV-2 high- and low-viral-load ROIs), while shapes show the tissue types (alveolar, large airway, and vascular regions).

(B) Similarity score distribution when compared with a tissue-specific (278 alveolar, 45 large airway, and 34 vascular ROIs) healthy reference gene profile (ns, non-significant, *p $\leq$ 0.05, **p $\leq$ 0.01, ***p $\leq$ 0.001, and ****p $\leq$ 0.0001).

(legend continued on next page)

SFTPC) associated with type 1 and type 2 pneumocytes as well as an enrichment for genes associated with basal cells (TP63) and club cells (SCGB1A1), and several immune markers (e.g., HLA-B, HLA-E, p all < 0.05, mixed effects model) in SARS-CoV-2-high patients compared with both normal and SARS-CoV-2-low patients. Ternary plots of a combined analysis of normal, SARS-CoV-2-high and -low tissues—where transcripts are projected away from the center based on their marginal means—revealed upregulation of several genes enriched in each set of lung tissues. Enrichments included SFTPA1, SFTPB, and SFTPC (alveolar epithelial cell markers) in normal lungs, CLU (lung injury and repair) and S100A9 (enriched in activated macrophages) in SARS-CoV-2-low lungs, and TP63 (basal cell), ID1 (upregulated and a key regulator of lung injury and repair), and interferon-regulated genes including IFI6, IFI27, ISG15, and LY6E in SARS-CoV-2-high lungs (Figures 4C and 4D). Enrichment of interferon-stimulated genes was observed only in SARS-CoV-2-high samples, which corresponds to early stages of the infection, by timing of disease onset and histopathology (Figures S1D and S1E). Moreover, we observed enrichments of CASP3 and ID1, suggesting ongoing cellular injury and repair responses in SARS-CoV-2-high patients. In contrast, we find an enrichment of several markers of pulmonary fibrosis (CLU, COL1A1, COL1A2, and COL3A1) in SARS-CoV-2-low patients (these correspond to the later stages of infection), indicating that there are two distinct stages of infection.[15]

We also examined the spatial transcriptome data for differences between the high- and low-SARS-CoV-2- and the IAV-infected lung samples. This analysis revealed a significant enrichment of THBS1 and NR4A1 in the influenza samples, which are both genes that have been shown to be engaged in response to influenza-induced lung injury (Figure S4B). When lung tissues from SARS-CoV-2-high and -low samples were compared with those from nonviral ARDS, enrichment for S100A8 and S100A9 was observed, which is consistent with the significant enrichment of neutrophils in these samples and was previously suggested to be a driver of COVID-19 pathogenesis (Figure S4A).[3,16] Importantly, an enrichment for genes involved in lung injury and repair (ID1), as well as those involved in type I interferon responses including IFI6, IFI27, ISG15, and LY6E in SARS-CoV-2-high lungs, is observed even when compared with IAV infection of the lung and nonviral lung injury. Similarly, we find enrichment of several markers of pulmonary fibrosis (CLU, COL1A1, COL1A2, and COL3A1) in SARS-CoV-2-low samples as compared with nonviral and viral ARDS samples, exemplifying the profound lung injury and fibrosis during later stages of COVID-19.

COVID-19-specific heterogeneity and spatial tropism in the lung

Regardless of specific lung-tissue types, the expression profiles and respective cell-type proportions were enough to distinguish and cluster normal versus COVID-19 (high and low SARS-CoV-2 viral loads) lung ROIs (Figure 5A), which indicates that the SARS-CoV-2 infection is altering the cellular interaction landscape and composition of the lung tissue. To gain additional insights into the heterogeneity and spatial tropism of lung infection independent of ROI origins, we assessed how each ROI compares with the typical healthy tissue. Some ROIs (especially of alveolar regions) in COVID-19 showed significantly diminished similarity to those of healthy lungs (Figures 5B, S5A, and S5B). Interestingly, the loss of this similarity did not result in convergence to other tissue types we observed (i.e., loss of alveolar similarity score did not increase the vascular or large airway scores). To evaluate the cause of this loss of similarities, we discovered genes attributing disease- and tissue-specific clustering and identified 35 genes with the highest contrast (Figure 5C). For example, some of these genes were related to regulatory T cell differentiation in COVID-19 high- and low-viral-load alveolar tissues (PLK1, ATM) and IL6/JAK/STAT3 signaling in SARS-CoV-2-low vascular regions (SOCS1, CXCL11; Table S3). In addition to the known molecular responses to COVID-19, these genes can be used as a set to locate infection response within the lung and disease states.

To pinpoint specific changes in different lung tissue types, count estimates of 15 distinct cell types were imputed based on gene expression profiles from the Human Cell Atlas (HCA) adult lung dataset, including neutrophil profiles derived from single-nucleus RNA-seq (snRNA-seq) of lung tumors (see STAR Methods). Consistent with other studies,[17,18] we observed that COVID-19 was associated with an increase in tissue-infiltrating immune cells, including T cells, natural killer (NK) cells, monocytes, and macrophages (Figures 5D and S5C). Some immune cell types, such as monocytes, NK cells, and regulatory T cells, showed a statistically significant increase only in the SARS-CoV-2-high conditions. Also, while fibroblasts and endothelial cells increased in both high- and low-viral-load samples (52% and 65% increase, respectively), type 1 and type 2 alveolar epithelial cell proportions decreased (26% and 16% decrease, respectively), reflecting the ongoing tissue remodeling or selective epithelial cell death induced by infection (Figure S5C). We validated these findings by comparing the expression levels with cell-type-specific COVID-19 gene signatures derived from snRNA-seq,[19] which showed enrichments across different cell types (Figure 5E; Table S3). Using an orthogonal approach, we stained the lung tissues with Masson's trichrome and observed a statistically significant increase ($p < 0.01$) in cellular collagen-rich areas, confirming the increase in lung fibroblasts (Figure S3A).

Given the observed changes in cell proportions that are induced during SARS-CoV-2 infection, we next examined the impact on the co-occurrence of these changes as a metric of intrapulmonary cellular heterogeneity (Figure 6). Pairwise correlations of all detected cell types under five different conditions

(C) Genes identifying disease- and tissue-specific conditions.

(D) Cell-type proportions in normal versus COVID-19. The median and quartiles are noted by the box plot inside. p value two-tailed t tests were done to compare the means (ns, *$p \leq 0.05$, **$p \leq 0.01$, ***$p \leq 0.001$, and ****$p \leq 0.0001$).

(E) Enrichment of cell type- and COVID-19-specific gene signatures from 11 patients across 247 ROIs.

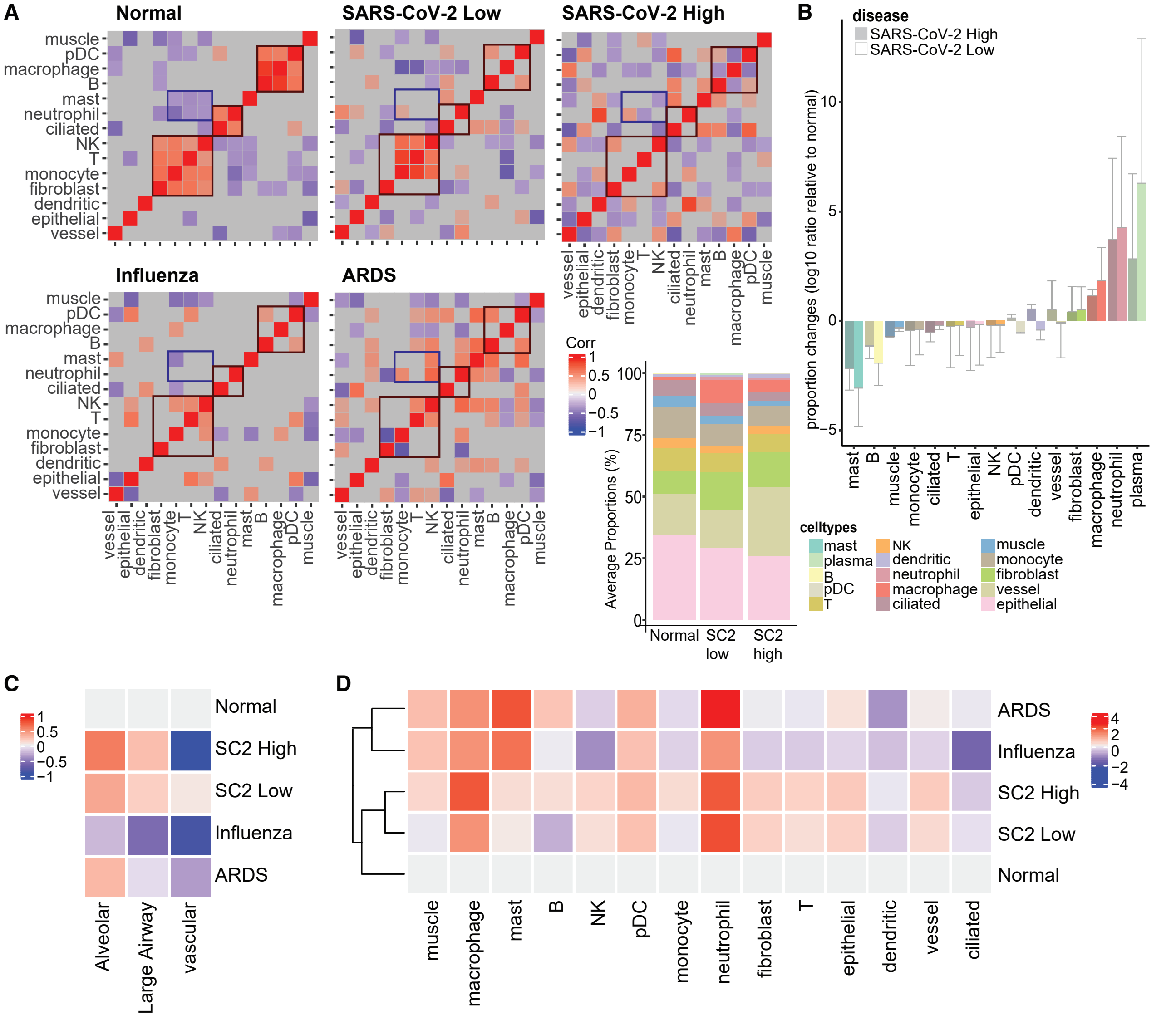

Figure 6. Cellular tropism and heterogeneity in response to COVID-19

(A) Correlation matrix of pairwise cell-type correlations. Statistically insignificant correlations were not displayed (gray area, $p < 0.05$); from 16 patients across 357 ROIs in total, as shown in Figure 1A.

(B) Average proportion changes of the cell types relative to normal. The cell types were ordered by average increase in proportions across 357 ROIs. Error bars indicate 0.5*SD. On the left, stacked bar plot depicts the overall proportions by conditions.

(C and D) Entropy estimates of the (C) tissue types and (D) cell types within the normal (n = 3, 64 ROIs), SARS-CoV-2-high (n = 4, 86 ROIs) and -low (n = 4, 97 ROIs), influenza (n = 2, 46 ROIs), and ARDS (n = 3, 67 ROIs) conditions.

(SARS-CoV-2 high and low, IAV, nonviral ARDS, and control) were calculated and visualized (Figure 6A). In normal lungs, we observed three clear cellular correlation clusters: (1) monocytes, fibroblasts, T cells, and NK cells, (2) neutrophils and ciliated cells, and (3) plasmacytoid dendritic cells (pDCs), macrophages, and B cells. While perturbations of these cellular clusters were observed across all injury conditions, the NK cell-T cell correlation was lost only in the SARS-CoV-2 high-viral-load patients and was not present in low-viral-load patients, with the low viral load corresponding to the later stages of infection (Figures 6A and 6B). We then quantified the correlation differences in the lungs' cellular landscapes between SARS-CoV-2-high and -low patients, which indicated that the greatest changes were in the monocyte-T cell correlations and dendritic-neutrophil correlations (Figure S6A), further supporting the view that SARS-CoV-2-specific T cell activity may be disrupted. Of note, it may be possible that the changes in correlation reflect both the generation of long-term memory and T cell-mediated killing of infected epithelial cells.[20]

To compare changes across the disease states (COVID-19, influenza, ARDS), we visualized the average proportions of each cell type. Macrophage and neutrophil population levels

were found to be much higher in the COVID-19 lungs (both high and low viral loads), while T cell, monocyte, and epithelial cell numbers were much lower than normal. The entropy estimates of the given cell types (Figure 6C), which are a function of variance, showed that macrophages and neutrophils commonly displayed increased heterogeneity (across ROIs and patients) across all injury conditions. Entropy in fibroblast, epithelial, vessel, and T cell populations was greater in SARS-CoV-2-high and -low lung tissue than in IAV infection and ARDS (121.5%, 104.7%, 111.9%, and 124.7% changes, respectively, when compared with the average of SARS-CoV-2 high/low and that of IAV/ARDS; Figure 6D), suggesting an increase in cellular heterogeneity. When measuring and comparing cellular tropism within the tissue, nearly all COVID-19-positive ROIs showed an increase in heterogeneity of the cell populations, with the sole exception being vascular regions with high COVID-19 (Figure 6D). The decrease in heterogeneity in the vascular regions of high-viral-load conditions is mainly from the decrease of fibroblasts and epithelial, T, and NK cells (Figure S6B). The signals from B cells are also specific to large airway tissues, and this observation is consistent with the cell fraction increase in large airway ROIs of COVID-19 samples (Figure S6B). Such changes in heterogeneity are related to specific damages and tissue dysregulations. Single-sample GSEA (ssGSEA) of macrophage, neutrophil, and T cell regulatory pathways showed enrichment in COVID-19, even when compared with influenza and ARDS, including macrophage activation and apoptotic process (1.3- and 2.8-fold increase in averaged ssGSEA scores relative to normal, with p values of 2.91×10^{-8} and 0.001) in patients with high viral loads (Figures S6C and S6E).

DISCUSSION

In this study, we established a clinical analytical pipeline to collect and examine autopsy samples to elucidate and compare the spatial transcriptional landscape induced by SARS-CoV-2, IAV, and nonviral ARDS. By combining transcriptional profiles of 39 patient autopsy tissues from heart, liver, lung, kidney, and lymph nodes, we presented body-wide transcriptome changes in response to COVID-19. Across all tissues, we found system-wide disruptions of cellular and transcriptional pathways and matched the lung data with spatial protein and expression profiling (GeoMx, across 357 tissue sections from 16 representative patient lung samples). We also identified tissue-compartment-specific damage (alveolar, vascular, and large airway compartments within the lung tissue) and the loss of tissue type identity caused by the SARS-CoV-2 infection, which correlated with viral loads (high versus low) and the clinical course of infection.

Patient lung tissue samples containing significant levels of SARS-CoV-2 showed enrichment for genes related to a variety of immune markers specific to certain immune cells and lung injuries as well as for interferon-stimulated genes (e.g., IFI27, IFITM1, and LY6E) and macrophage activation (S100A9, TYMP, and SERPING1). In contrast, patient lung tissue samples containing low levels of SARS-CoV-2 RNA show enrichment for COL1A1 and other markers of pulmonary fibrosis. Compared with other viral-related diseases (influenza), COVID-19 tissue samples still show significant enrichment for genes involved in lung injury and repair, interferon-signaling genes, and pulmonary fibrosis. Of note, COVID-19 (high-viral-load samples), influenza, and ARDS each show differential HLA-B and -C expressions, which are known mediators of NK and T cell activation[21,22] and which can mediate host risk of infection; one example is enrichment for HLA-DRB5, whose expression and specific gene polymorphisms are associated with pulmonary fibrosis and severity.[23,24] Compared across different disease types, all diseases, COVID-19 (low viral load), influenza, and ARDS showed enrichment for DMBT1, a gene known to be upregulated and dysregulated in pulmonary injury and fibrosis.[25,26] Virus-related diseases (SARS-CoV-2 high viral load and influenza) in particular showed significant changes on the expression of lung epithelial-cell-related transcripts (i.e., ACTB, C1R, and FN1); such changes are known markers of lung-injury gene signatures.[27,28]

The spatial analysis platform (Nanostring GeoMx) enabled us to investigate the impact of the disease by incorporating cellular and spatial organization. Consistent with recent reports from bulk cellular profiling, we observed an increase in immune cell types and fibroblasts in COVID-19 but a decrease in alveolar epithelial cells.[10] In SARS-CoV-2 low-viral-load tissues, the proportions of some immune cells (i.e., monocytes, NK cells, or regulatory T cells) were normal, but fibroblasts and vessel cells still exhibited an increase similar to those observed in the high-viral-load samples. Some of these cell types form a “cellular correlation cluster,” and these co-occurrence clusters of cellular changes are uniquely disrupted in COVID-19 (relative to influenza and ARDS), particularly in the COVID-19 high-viral-load sample group. While macrophages and neutrophils showed an increase in entropy across all lung-related injury conditions, NK and T cells showed an increase only in COVID-19 samples. While few studies have interrogated the tissue environments, multiple studies have examined the changes occurring during COVID-19 infection in the peripheral blood and have identified poor T cell responses and T cell dysregulation.[27,29–33] Together, these findings highlight the robust and dynamic nature of SARS-CoV-2 engagement with tissue homeostatic processes and that the stage of COVID-19 infection impacts the pathophysiological landscape of the lung.

When these spatial transcriptomics data were compared to the multi-organ bulk RNA-seq data from the autopsy issues, confirmatory as well as additional signatures of COVID-19 disease were found. First, fibroblasts and immune cells (i.e., macrophages) were increased in most tissue types, while tissue-specific cell types, such as alveolar epithelial types 1 and 2 cells in the lung and cardiomyocytes in the heart, showed a decrease in COVID-19 relative to controls. Increases in fibroblasts, endothelial cells, and immune cells may be impacted by a variety of immune activations within each organ, particularly as a long-term response to the infection. This observation may also be related to the decrease in characteristic transcriptomic signatures of the main cell type within each organ, which may contribute to the morbidity and mortality of COVID-19. For example, the reduction in the cardiomyocyte cell fraction correlated with a reduction in several transcripts encoding sarcomeric and contractile proteins (in both high and low viral loads),[34,35] representing a persistent transcriptional perturbation and potentially long-term, cardiac-specific impact of COVID-19.

These data support the view of both tissue-specific and time-dependent biological responses to the stages of SARS-CoV-2 infection, and this is buttressed by orthogonal data. For example, DEGs observed from NP swabs showed a high correlation to tissue-specific DEGs in the early stages of infection but very little correlation with later infection. The observed correlations likely reveal monocyte migration and infiltration into tissues during SARS-CoV-2 infection, as seen by others.[36] It has also been reported that monocyte depletion/migration is associated with kidney disease, inducing lupus-like symptoms,[37] which could potentially explain the correlation with kidney tissue. For lymph nodes, there exists evidence in the literature that SARS-CoV-2 will impact the lymph nodes at an early stage of infection, potentially causing T cell lymphopenia and possibly being responsible for focal necrosis seen in the lymph nodes.[38] Nonetheless, the lung, heart, and lymph nodes were the tissues most disrupted by infection.

Overall, these data represent one of the largest autopsy series of COVID-19 disease and synthesize several orthogonal methods, including bulk transcriptomics, digital spatial transcriptomics, multiple imaging technologies, and computational analysis, to build a map of SARS-CoV-2 pathophysiology. Given the ability to combine bulk transcriptomics data from multiple organ types, we find organ-specific changes to immune responses and the loss of tissue functions unique to COVID-19 disease, which can help additional studies and methods for mitigating the systemic damage caused by the SARS-CoV-2 virus across the body.

Limitations of the study

While the size of the cohort we used for spatial profiling is smaller than that used for bulk transcriptomics, we believe an in-depth characterization of patient tissues is crucial in directly validating and supporting the findings from cellular and animal models.[39,40] Spatial profiling technology captures key aspects of COVID-19 that bulk data cannot, including the locations of transcriptional and cellular changes caused by the disease (especially in late infection) and spatial heterogeneity of cell types. The compartment-specific COVID-19 gene signatures would need further validation (e.g. chromatin states and additional validation), as we rely on computational methodologies and deconvolution techniques, and could also benefit from matched profiling of peripheral blood,[41] examination of strain type,[42] and also differences in vaccination status.[43] Nonetheless, this molecular map of COVID-19 represents a needed cellular and molecular atlas for the community, which can inform future studies into COVID-19 progression and SARS-CoV-2 pathology.

STAR★METHODS

Detailed methods are provided in the online version of this paper and include the following:

- KEY RESOURCES TABLE
- RESOURCE AVAILABILITY
  - Lead contact
  - Materials availability
  - Data and code availability
- EXPERIMENTAL MODEL AND SUBJECT DETAILS
  - IRB statement
  - Patient sample collection
- METHOD DETAILS
  - qRT-PCR
  - RNA-seq analysis
  - Spatial transcriptomics analysis
- QUANTIFICATION AND STATISTICAL ANALYSIS
  - RNA-seq analysis
  - GeoMx transcriptomic data normalization and quantification

SUPPLEMENTAL INFORMATION

Supplemental information can be found online at https://doi.org/10.1016/j.xcrm.2022.100522.

ACKNOWLEDGMENTS

We thank the patients, their families, and healthcare workers fighting the COVID-19 pandemic. This work was supported by the NCI (R01CA234614), the NIAID (2R01AI107301), and the NIDDK (R01DK121072 and 1RO3DK117252) to the Department of Medicine, Weill Cornell Medicine (R.E.S.). R.E.S. is supported as an Irma Hirschl Trust Research Award Scholar. A.B. is supported by supplemental funds for COVID-19 research from the Translational Research Institute for Space Health (TRISH) through NASA Cooperative Agreement NNX16AO69A (T-0404), and further funding was provided by KBR, Inc. Sequencing of some samples was performed at the New York Genome Center (NYGC) as part of the COVID-19 Genomic Research Network (CGRN) with funds generously provided by NYGC donors. A.F.R. is supported by NCI T32CA203702 grant. We would like to thank the Epigenomics Core Facility at Weill Cornell Medicine, the Scientific Computing Unit (SCU), as well as the Starr Cancer Consortium (I9-A9-071 and I13-0052), the NIH (R21AI129851, R01MH117406, R01CA249054, R01AI151059, P01CA214274, and U01DA053941), the Leukemia and Lymphoma Society (LLS; MCL7001-18, LLS 9238-16, and LLS-MCL7001-18), Testing for America (501c3), the OpenCovidScreen Foundation, the Rockefeller Foundation, Igor Tulchinsky, the WorldQuant Foundation, Bill Ackman, Olivia Flatto, the Pershing Square Foundation, and Ken Griffin and Citadel.

AUTHOR CONTRIBUTIONS

R.E.S., A.S., and C.E.M. conceived and designed the experiments. J.P. performed spatial transcriptomic analyses and statistical investigation, with contributions from T.H., S.W., Y.K., J. Reeves, A.R.F., and C.M.R. C. Meydan, J.F., and J.P. performed the RNA-seq bioinformatics analyses and statistical investigations, with contributions from D.C.D. A.C.B. was involved in autopsy tissue procurement, pathology evaluation, GeoMx ROI selection, and trichrome quantitation. R.E.S. processed and analyzed samples and clinical data, with contributions from Y.B. and V.C. D.J.B., C. Mozsary, E.E.A., M.M., S.L., M.S., A.M.M., I.H., S.W., A.C., P.V., M.S., L.F.W., M.C., H.R., and N.P.T. organized and worked with NP swabs and data. H.G., S.F., A.C., M.C.Z., S.G., A.B., D.T., A.S.-B., U.S., E.S.W., and J.S. performed the RNA-seq and worked on data analysis, interpretation, and figures, with contributions from M.I., A.S., J. Rosiene, S. Salvatore, S. Shapira, A.J.K., and O.E. All authors discussed the results and contributed to the final manuscript.

DECLARATION OF INTERESTS

O.E. is scientific adviser and equity holder in Freenome, Owkin, Volastra Therapeutics, and OneThree Biotech. R.E.S. is on the scientific advisory board of Miromatrix, Inc., and is a consultant and speaker for Alnylam, Inc. L.S. is a scientific co-founder and paid consultant. C.M. and E.E.A. are consultants for Onegevity Health. C.E.M. is a co-founder of Biotia and Onegevity Health and

an advisor to Nanostring. T.H., S.W., Y.K., and J.R. are employees of Nanostring, Inc. All other authors declare no competing interests.

Received: July 7, 2021
Revised: December 22, 2021
Accepted: January 16, 2022
Published: January 24, 2022

REFERENCES

1. He, X., Lau, E.H.Y., Wu, P., Deng, X., Wang, J., Hao, X., Lau, Y.C., Wong, J.Y., Guan, Y., Tan, X., et al. (2020). Temporal dynamics in viral shedding and transmissibility of COVID-19. Nat. Med. *26*, 672–675. https://doi.org/10.1038/s41591-020-0869-5.
2. Eastin, C., and Eastin, T. (2020). Clinical Characteristics of Coronavirus Disease 2019 in China: Guan W, Ni Z, Hu Y, et al. N Engl J Med. 2020 Feb 28 [Online ahead of print]. J. Emerg. Med. *58*, 711–712. https://doi.org/10.1016/j.jemermed.2020.04.004.
3. Guo, Q., Zhao, Y., Li, J., Liu, J., Yang, X., Guo, X., Kuang, M., Xia, H., Zhang, Z., Cao, L., et al. (2021). Induction of alarmin S100A8/A9 mediates activation of aberrant neutrophils in the pathogenesis of COVID-19. Cell Host Microbe *29*, 222–235.e4. https://doi.org/10.1016/j.chom.2020.12.016.
4. Butler, D., Mozsary, C., Meydan, C., Foox, J., Rosiene, J., Shaiber, A., Danko, D., Afshinnekoo, E., MacKay, M., Sedlazeck, F.J., et al. (2021). Shotgun transcriptome, spatial omics, and isothermal profiling of SARS-CoV-2 infection reveals unique host responses, viral diversification, and drug interactions. Nat. Commun. *12*, 1660. https://doi.org/10.1038/s41467-021-21361-7.
5. Lansbury, L., Lim, B., Baskaran, V., and Lim, W.S. (2020). Co-infections in people with COVID-19: a systematic review and meta-analysis. J. Infect. *81*, 266–275. https://doi.org/10.1016/j.jinf.2020.05.046.
6. Lei, X., Dong, X., Ma, R., Wang, W., Xiao, X., Tian, Z., Wang, C., Wang, Y., Li, L., Ren, L., et al. (2020). Activation and evasion of type I interferon responses by SARS-CoV-2. Nat. Commun. *11*, 3810. https://doi.org/10.1038/s41467-020-17665-9.
7. Sharifipour, E., Shams, S., Esmkhani, M., Khodadadi, J., Fotouhi-Ardakani, R., Koohpaei, A., Doosti, Z., and EJ Golzari, S. (2020). Evaluation of bacterial co-infections of the respiratory tract in COVID-19 patients admitted to ICU. BMC Infect. Dis. *20*, 646. https://doi.org/10.1186/s12879-020-05374-z.
8. Wang, L., Yu, P., Zhou, B., Song, J., Li, Z., Zhang, M., Guo, G., Wang, Y., Chen, X., Han, L., and Hu, S. (2020). Single-cell reconstruction of the adult human heart during heart failure and recovery reveals the cellular landscape underlying cardiac function. Nat. Cell Biol. *22*, 108–119. https://doi.org/10.1038/s41556-019-0446-7.
9. Kim, D., Lee, J.-Y., Yang, J.-S., Kim, J.W., Kim, V.N., and Chang, H. (2020). The architecture of SARS-CoV-2 transcriptome. Cell *181*, 914–921.e10. https://doi.org/10.1016/j.cell.2020.04.011.
10. Reyfman, P.A., Walter, J.M., Joshi, N., Anekalla, K.R., McQuattie-Pimentel, A.C., Chiu, S., Fernandez, R., Akbarpour, M., Chen, C.-I., Ren, Z., et al. (2019). Single-cell transcriptomic analysis of human lung provides insights into the pathobiology of pulmonary fibrosis. Am. J. Respir. Crit. Care Med. *199*, 1517–1536. https://doi.org/10.1164/rccm.201712-2410OC.
11. Felice, D., Kathie-Anne, W., Yongli, X., Zong-Mei, S., Kelsey, S., Jaekeun, P., Sebastian, G., Angela, R.L., Kaitlyn, S., Heather, K., et al. (2021). Lung epithelial and endothelial damage, loss of tissue repair, inhibition of fibrinolysis, and cellular senescence in fatal COVID-19. Sci. Transl. Med. *13*, eabj7790. https://doi.org/10.1126/scitranslmed.abj7790.
12. Lindner, D., Fitzek, A., Bräuninger, H., Aleshcheva, G., Edler, C., Meissner, K., Scherschel, K., Kirchhof, P., Escher, F., Schultheiss, H.-P., et al. (2020). Association of cardiac infection with SARS-CoV-2 in confirmed COVID-19 autopsy cases. JAMA Cardiol. *5*, 1281–1285. https://doi.org/10.1001/jamacardio.2020.3551.
13. Babapoor-Farrokhran, S., Gill, D., Walker, J., Rasekhi, R.T., Bozorgnia, B., and Amanullah, A. (2020). Myocardial injury and COVID-19: possible mechanisms. Life Sci. *253*, 117723.
14. Lee, S., Yu, Y., Trimpert, J., Benthani, F., Mairhofer, M., Richter-Pechanska, P., Wyler, E., Belenki, D., Kaltenbrunner, S., Pammer, M., et al. (2021). Virus-induced senescence is a driver and therapeutic target in COVID-19. Nature *599*, 283–289. https://doi.org/10.1038/s41586-021-03995-1.
15. Desai, N., Neyaz, A., Szabolcs, A., Shih, A.R., Chen, J.H., Thapar, V., Nieman, L.T., Solovyov, A., Mehta, A., Lieb, D.J., et al. (2020). Temporal and spatial heterogeneity of host response to SARS-CoV-2 pulmonary infection. Nat. Commun. *11*, 6319. https://doi.org/10.1038/s41467-020-20139-7.
16. Silvin, A., Chapuis, N., Dunsmore, G., Goubet, A.-G., Dubuisson, A., Derosa, L., Almire, C., Hénon, C., Kosmider, O., Droin, N., et al. (2020). Elevated calprotectin and abnormal myeloid cell subsets discriminate severe from mild COVID-19. Cell *182*, 1401–1418.e18. https://doi.org/10.1016/j.cell.2020.08.002.
17. Chevrier, S., Zurbuchen, Y., Cervia, C., Adamo, S., Raeber, M.E., de Souza, N., Sivapatham, S., Jacobs, A., Bachli, E., Rudiger, A., et al. (2021). A distinct innate immune signature marks progression from mild to severe COVID-19. Cell Rep. Med. *2*, 100166. https://doi.org/10.1016/j.xcrm.2020.100166.
18. Liao, M., Liu, Y., Yuan, J., Wen, Y., Xu, G., Zhao, J., Cheng, L., Li, J., Wang, X., Wang, F., et al. (2020). Single-cell landscape of bronchoalveolar immune cells in patients with COVID-19. Nat. Med. *26*, 842–844. https://doi.org/10.1038/s41591-020-0901-9.
19. Melms, J.C., Biermann, J., Huang, H., Wang, Y., Nair, A., Tagore, S., Katsyv, I., Rendeiro, A.F., Amin, A.D., Schapiro, D., et al. (2021). A molecular single-cell lung atlas of lethal COVID-19. Nature *595*, 114–119. https://doi.org/10.1038/s41586-021-03569-1.
20. Channappanavar, R., Zhao, J., and Perlman, S. (2014). T cell-mediated immune response to respiratory coronaviruses. Immunol. Res. *59*, 118–128. https://doi.org/10.1007/s12026-014-8534-z.
21. Blais, M.-E., Dong, T., and Rowland-Jones, S. (2011). HLA-C as a mediator of natural killer and T-cell activation: spectator or key player? Immunology *133*, 1–7. https://doi.org/10.1111/j.1365-2567.2011.03422.x.
22. Johnson, D.R. (2000). Differential expression of human major histocompatibility class I loci: HLA-A, -B, and -C. Hum. Immunol. *61*, 389–396. https://doi.org/10.1016/S0198-8859(99)00186-X.
23. Fang, C., Huang, H., Feng, Y., Zhang, Q., Wang, N., Jing, X., Guo, J., Feriainc, M., and Xu, Z. (2021). Whole-exome sequencing identifies susceptibility genes and pathways for idiopathic pulmonary fibrosis in the Chinese population. Sci. Rep. *11*, 1443. https://doi.org/10.1038/s41598-020-80944-4.
24. Corvol, H., Blackman, S.M., Boëlle, P.-Y., Gallins, P.J., Pace, R.G., Stonebraker, J.R., Accurso, F.J., Clement, A., Collaco, J.M., Dang, H., et al. (2015). Genome-wide association meta-analysis identifies five modifier loci of lung disease severity in cystic fibrosis. Nat. Commun. *6*, 8382. https://doi.org/10.1038/ncomms9382.
25. Tian, Y., Li, H., Gao, Y., Liu, C., Qiu, T., Wu, H., Cao, M., Zhang, Y., Ding, H., Chen, J., and Cai, H. (2019). Quantitative proteomic characterization of lung tissue in idiopathic pulmonary fibrosis. Clin. Proteomics *16*, 6. https://doi.org/10.1186/s12014-019-9226-4.
26. Kılıç, A., Ameli, A., Park, J.-A., Kho, A.T., Tantisira, K., Santolini, M., Cheng, F., Mitchel, J.A., McGill, M., O'Sullivan, M.J., et al. (2020). Mechanical forces induce an asthma gene signature in healthy airway epithelial cells. Sci. Rep. *10*, 966. https://doi.org/10.1038/s41598-020-57755-8.
27. Rendeiro, A.F., Casano, J., Vorkas, C.K., Singh, H., Morales, A., DeSimone, R.A., Ellsworth, G.B., Soave, R., Kapadia, S.N., Saito, K., et al. (2021). Profiling of immune dysfunction in COVID-19 patients allows early prediction of disease progression. Life Sci. Alliance *4*, e202000955. https://doi.org/10.26508/lsa.202000955.

28. Margaroli, C., Benson, P., Sharma, N.S., Madison, M.C., Robison, S.W., Arora, N., Ton, K., Liang, Y., Zhang, L., Patel, R.P., and Gaggar, A. (2021). Spatial mapping of SARS-CoV-2 and H1N1 lung injury identifies differential transcriptional signatures. Cell Rep. Med. *2*, 100242. https://doi.org/10.1016/j.xcrm.2021.100242.

29. Delorey, T.M., Ziegler, C.G.K., Heimberg, G., Normand, R., Yang, Y., Segerstolpe, Å., Abbondanza, D., Fleming, S.J., Subramanian, A., Montoro, D.T., et al. (2021). COVID-19 tissue atlases reveal SARS-CoV-2 pathology and cellular targets. Nature *595*, 107–113. https://doi.org/10.1038/s41586-021-03570-8.

30. Takahashi, T., Ellingson, M.K., Wong, P., Israelow, B., Lucas, C., Klein, J., Silva, J., Mao, T., Oh, J.E., Tokuyama, M., et al. (2020). Sex differences in immune responses that underlie COVID-19 disease outcomes. Nature *588*, 315–320. https://doi.org/10.1038/s41586-020-2700-3.

31. Lucas, C., Wong, P., Klein, J., Castro, T.B.R., Silva, J., Sundaram, M., Ellingson, M.K., Mao, T., Oh, J.E., Israelow, B., et al. (2020). Longitudinal analyses reveal immunological misfiring in severe COVID-19. Nature *584*, 463–469. https://doi.org/10.1038/s41586-020-2588-y.

32. Rydyznski Moderbacher, C., Ramirez, S.I., Dan, J.M., Grifoni, A., Hastie, K.M., Weiskopf, D., Belanger, S., Abbott, R.K., Kim, C., Choi, J., et al. (2020). Antigen-specific adaptive immunity to SARS-CoV-2 in acute COVID-19 and associations with age and disease severity. Cell *183*, 996–1012.e19. https://doi.org/10.1016/j.cell.2020.09.038.

33. Rendeiro, A.F., Ravichandran, H., Bram, Y., Chandar, V., Kim, J., Meydan, C., Park, J., Foox, J., Hether, T., Warren, S., et al. (2021). The spatial landscape of lung pathology during COVID-19 progression. Nature *593*, 564–569. https://doi.org/10.1038/s41586-021-03475-6.

34. Basso, C., Leone, O., Rizzo, S., De Gaspari, M., van der Wal, A.C., Aubry, M.-C., Bois, M.C., Lin, P.T., Maleszewski, J.J., and Stone, J.R. (2020). Pathological features of COVID-19-associated myocardial injury: a multicentre cardiovascular pathology study. Eur. Heart J. *41*, 3827–3835. https://doi.org/10.1093/eurheartj/ehaa664.

35. Siddiq, M.M., Chan, A.T., Miorin, L., Yadaw, A.S., Beaumont, K.G., Kehrer, T., White, K.M., Cupic, A., Tolentino, R.E., Hu, B., et al. (2020). Physiology of cardiomyocyte injury in COVID-19. Medrxiv. https://doi.org/10.1101/2020.11.10.20229294.

36. Boumaza, A., Gay, L., Mezouar, S., Bestion, E., Diallo, A.B., Michel, M., Desnues, B., Raoult, D., La Scola, B., Halfon, P., et al. (2021). Monocytes and macrophages, targets of severe acute respiratory Syndrome coronavirus 2: the clue for coronavirus disease 2019 immunoparalysis. J. Infect. Dis. *224*, 395–406. https://doi.org/10.1093/infdis/jiab044.

37. Kuriakose, J., Redecke, V., Guy, C., Zhou, J., Wu, R., Ippagunta, S.K., Tillman, H., Walker, P.D., Vogel, P., and Häcker, H. (2019). Patrolling monocytes promote the pathogenesis of early lupus-like glomerulonephritis. J. Clin. Invest. *129*, 2251–2265. https://doi.org/10.1172/JCI125116.

38. Merad, M., and Martin, J.C. (2020). Pathological inflammation in patients with COVID-19: a key role for monocytes and macrophages. Nat. Rev. Immunol. *20*, 355–362. https://doi.org/10.1038/s41577-020-0331-4.

39. Yang, L., Liu, S., Liu, J., Zhang, Z., Wan, X., Huang, B., Chen, Y., and Zhang, Y. (2020). COVID-19: immunopathogenesis and immunotherapeutics. Signal. Transduct Target Ther. *5*, 128. https://doi.org/10.1038/s41392-020-00243-2.

40. Frere JJ, Serafini RA, Pryce KD, Golynker I, Panis M, Zimering J, Horiuchi S, Hoagland DA, Moeller R, Oishi K, et al A Molecular Basis of Long COVID-19.

41. Ng, L., Granados, A.C., Santos, Y.A., Servellita, V., Goldgof, G.M., Meydan, C., Sotomayor-Gonzalez, A., Levine, A.G., Balcerek, J., Han, L.M., et al. (2021). A diagnostic host response biosignature for COVID-19 from RNA profiling of nasal swabs and blood. Sci. Adv. *7*, eabe5984. https://doi.org/10.1126/sciadv.abe5984.

42. Alpert, T., Brito, A.F., Lasek-Nesselquist, E., Rothman, J., Valesano, A.L., MacKay, M.J., Petrone, M.E., Breban, M.I., Watkins, A.E., Vogels, C.B.F., et al. (2021). Early introductions and transmission of SARS-CoV-2 variant B.1.1.7 in the United States. Cell *184*, 2595–2604.e13. https://doi.org/10.1016/j.cell.2021.03.061.

43. Kissler, S.M., Fauver, J.R., Mack, C., Tai, C.G., Breban, M.I., Watkins, A.E., Samant, R.M., Anderson, D.J., Metti, J., Khullar, G., et al. (2021). Viral dynamics of SARS-CoV-2 variants in vaccinated and unvaccinated persons. N. Engl. J. Med. https://doi.org/10.1056/NEJMc2102507.

44. Rother, N., Yanginlar, C., Lindeboom, R.G.H., Bekkering, S., van Leent, M.M.T., Buijsers, B., Jonkman, I., de Graaf, M., Baltissen, M., Lamers, L.A., et al. (2020). Hydroxychloroquine inhibits the trained innate immune response to interferons. Cell Rep. Med. *1*, 100146. https://doi.org/10.1016/j.xcrm.2020.100146.

45. Travaglini, K.J., Nabhan, A.N., Penland, L., Sinha, R., Gillich, A., Sit, R.V., Chang, S., Conley, S.D., Mori, Y., Seita, J., et al. (2020). A molecular cell atlas of the human lung from single-cell RNA sequencing. Nature *587*, 619–625. https://doi.org/10.1038/s41586-020-2922-4.

46. MacParland, S.A., Liu, J.C., Ma, X.-Z., Innes, B.T., Bartczak, A.M., Gage, B.K., Manuel, J., Khuu, N., Echeverri, J., Linares, I., et al. (2018). Single cell RNA sequencing of human liver reveals distinct intrahepatic macrophage populations. Nat. Commun. *9*, 4383. https://doi.org/10.1038/s41467-018-06318-7.

47. Stewart, B.J., Ferdinand, J.R., Young, M.D., Mitchell, T.J., Loudon, K.W., Riding, A.M., Richoz, N., Frazer, G.L., Staniforth, J.U., Braga, F.A., et al. (2019). Spatiotemporal immune zonation of the human kidney. Science *365*, 1461–1466. https://doi.org/10.1126/science.aat5031.

48. Liberzon, A., Subramanian, A., Pinchback, R., Thorvaldsdóttir, H., Tamayo, P., and Mesirov, J.P. (2011). Molecular signatures database (MSigDB) 3.0. Bioinformatics *27*, 1739–1740. https://doi.org/10.1093/bioinformatics/btr260.

49. Subramanian, A., Tamayo, P., Mootha, V.K., Mukherjee, S., Ebert, B.L., Gillette, M.A., Paulovich, A., Pomeroy, S.L., Golub, T.R., Lander, E.S., and Mesirov, J.P. (2005). Gene set enrichment analysis: a knowledge-based approach for interpreting genome-wide expression profiles. Proc. Natl. Acad. Sci. U S A *102*, 15545–15550. https://doi.org/10.1073/pnas.0506580102.

50. Kuleshov, M.V., Jones, M.R., Rouillard, A.D., Fernandez, N.F., Duan, Q., Wang, Z., Koplev, S., Jenkins, S.L., Jagodnik, K.M., Lachmann, A., et al. (2016). Enrichr: a comprehensive gene set enrichment analysis web server 2016 update. Nucleic Acids Res. *44*, W90–W97. https://doi.org/10.1093/nar/gkw377.

51. Ewels, P.A., Peltzer, A., Fillinger, S., Patel, H., Alneberg, J., Wilm, A., Garcia, M.U., Di Tommaso, P., and Nahnsen, S. (2020). The nf-core framework for community-curated bioinformatics pipelines. Nat. Biotechnol. *38*, 276–278. https://doi.org/10.1038/s41587-020-0439-x.

52. Andrews, S. (2010). FastQC: a quality control tool for high throughput sequence data.http://www.bioinformatics.babraham.ac.uk/projects/fastqc/.

53. Dobin, A., Davis, C.A., Schlesinger, F., Drenkow, J., Zaleski, C., Jha, S., Batut, P., Chaisson, M., and Gingeras, T.R. (2013). STAR: ultrafast universal RNA-seq aligner. Bioinformatics *29*, 15–21. https://doi.org/10.1093/bioinformatics/bts635.

54. Patro, R., Duggal, G., Love, M.I., Irizarry, R.A., and Kingsford, C. (2017). Salmon provides fast and bias-aware quantification of transcript expression. Nat. Methods *14*, 417–419. https://doi.org/10.1038/nmeth.4197.

55. Kovaka, S., Zimin, A.V., Pertea, G.M., Razaghi, R., Salzberg, S.L., and Pertea, M. (2019). Transcriptome assembly from long-read RNA-seq alignments with StringTie2. Genome Biol. *20*, 278. https://doi.org/10.1186/s13059-019-1910-1.

56. Li, H., and Durbin, R. (2009). Fast and accurate short read alignment with Burrows-Wheeler transform. Bioinformatics *25*, 1754–1760. https://doi.org/10.1093/bioinformatics/btp324.

57. Love, M.I., Huber, W., and Anders, S. (2014). Moderated estimation of fold change and dispersion for RNA-seq data with DESeq2. Genome Biol. *15*, 550. https://doi.org/10.1186/s13059-014-0550-8.

CellPress
OPEN ACCESS

58. Wang, X., Park, J., Susztak, K., Zhang, N.R., and Li, M. (2019). Bulk tissue cell type deconvolution with multi-subject single-cell expression reference. Nat. Commun. *10*, 380. https://doi.org/10.1038/s41467-018-08023-x.

59. Finotello, F., Rieder, D., Hackl, H., and Trajanoski, Z. (2019). Next-generation computational tools for interrogating cancer immunity. Nat. Rev. Genet. *20*, 724–746. https://doi.org/10.1038/s41576-019-0166-7.

60. Diedenhofen, B., and Musch, J. (2015). Cocor: a comprehensive solution for the statistical comparison of correlations. PLoS One *10*, e0121945.

61. Sapoval, N., Mahmoud, M., Jochum, M.D., Liu, Y., Elworth, R.A.L., Wang, Q., Albin, D., Ogilvie, H., Lee, M.D., Villapol, S., et al. (2020). Hidden genomic diversity of SARS-CoV-2: implications for qRT-PCR diagnostics and transmission. Biorxiv. https://doi.org/10.1101/2020.07.02.184481.

62. Merritt, C.R., Ong, G.T., Church, S.E., Barker, K., Danaher, P., Geiss, G., Hoang, M., Jung, J., Liang, Y., McKay-Fleisch, J., et al. (2020). Multiplex digital spatial profiling of proteins and RNA in fixed tissue. Nat. Biotechnol. *38*, 586–599. https://doi.org/10.1038/s41587-020-0472-9.

63. Danaher, P., Kim, Y., Nelson, B., Griswold, M., Yang, Z., Piazza, E., and Beechem, J.M. (2020). Advances in mixed cell deconvolution enable quantification of cell types in spatially-resolved gene expression data. bioRxiv. https://doi.org/10.1101/2020.08.04.235168.

64. Korotkevich, G., Sukhov, V., Budin, N., Shpak, B., Artyomov, M.N., and Sergushichev, A. (2021). Fast gene set enrichment analysis. bioRxiv. https://doi.org/10.1101/060012.

65. Benjamini, Y., and Hochberg, Y. (1995). Controlling the false discovery rate: a practical and powerful approach to multiple testing. J. R. Stat. Soc. Ser. B *57*, 289–300. https://doi.org/10.1111/j.2517-6161.1995.tb02031.x.

66. Friedman, J., Hastie, T., and Tibshirani, R. (2010). Regularization paths for generalized linear models via coordinate descent. J. Stat. Softw. *33*, 1–22.

67. Zilionis, R., Engblom, C., Pfirschke, C., Savova, V., Zemmour, D., Saatcioglu, H.D., Krishnan, I., Maroni, G., Meyerovitz, C.V., Kerwin, C.M., et al. (2019). Single-cell transcriptomics of human and mouse lung cancers reveals conserved myeloid populations across individuals and species. Immunity *50*, 1317–1334.e10. https://doi.org/10.1016/j.immuni.2019.03.009.

68. Kuznetsova, A., Brockhoff, P.B., and Christensen, R.H.B. (2017). lmerTest package: tests in linear mixed effects models. J. Stat. Softw. *82*, 1–26. https://doi.org/10.18637/jss.v082.i13.

69. Fang, Z., Martin, J., and Wang, Z. (2012). Statistical methods for identifying differentially expressed genes in RNA-Seq experiments. Cell Biosci. *2*, 26. https://doi.org/10.1186/2045-3701-2-26.

70. Jassal, B., Matthews, L., Viteri, G., Gong, C., Lorente, P., Fabregat, A., Sidiropoulos, K., Cook, J., Gillespie, M., Haw, R., et al. (2020). The reactome pathway knowledgebase. Nucleic Acids Res. *48*, D498–D503. https://doi.org/10.1093/nar/gkz1031.

71. Hänzelmann, S., Castelo, R., and Guinney, J. (2013). GSVA: gene set variation analysis for microarray and RNA-Seq data. BMC Bioinformatics *14*, 7. https://doi.org/10.1186/1471-2105-14-7.

72. Ruifrok, A., and Johnston, D. (2001). Quantification of histochemical staining by color deconvolution. Anal. Quant Cytol. Histol. *23*, 291–299.

73. Singh, U., Li, J., Seetharam, A., and Wurtele, E.S. (2021). pyrpipe: a Python package for RNA-Seq workflows. NAR Genomics Bioinforma *3*, lqab049. https://doi.org/10.1093/nargab/lqab049.

74. Singh, U., Hur, M., Dorman, K., and Wurtele, E.S. (2020). MetaOmGraph: a workbench for interactive exploratory data analysis of large expression datasets. Nucleic Acids Res. *48*, e23. https://doi.org/10.1093/nar/gkz1209.

75. Wood, D.E., Lu, J., and Langmead, B. (2019). Improved metagenomic analysis with Kraken 2. Genome Biol. *20*, 257. https://doi.org/10.1186/s13059-019-1891-0.

76. Grubaugh, N.D., Gangavarapu, K., Quick, J., Matteson, N.L., De Jesus, J.G., Main, B.J., Tan, A.L., Paul, L.M., Brackney, D.E., Grewal, S., et al. (2019). An amplicon-based sequencing framework for accurately measuring intrahost virus diversity using PrimalSeq and iVar. Genome Biol. *20*, 8. https://doi.org/10.1186/s13059-018-1618-7.

STAR★METHODS

KEY RESOURCES TABLE

REAGENT or RESOURCE	SOURCE	IDENTIFIER
**Antibodies**		
Immune Cell Profiling Panel (Core)	Nanostring Technologies, Inc	GMX-PROCONCT-HICP-12, Item 121300101, Lot# 0474026
10 Drug Target Panel	Nanostring Technologies, Inc	GMX-PROMODNCT-HIODT-12, Item 121300102, Lot# 0474029
Immune Activation Status Panel	Nanostring Technologies, Inc.	GMX-PROMODNCT-HIAS-12, Item 121300103, Lot# 0474032
Immune Cell Typing Panel	Nanostring Technologies, Inc	GMX-PROMODNCT-HICT-12, Item 121300104, Lot# 0474035
Cell Death Panel	Nanostring Technologies, Inc	GMX-PROMOD-NCTHCD-12, Lot# 0474050
MAPK Signaling Panel	Nanostring Technologies, Inc	GMX-PROMOD-NCTHMAPK-12, Lot# 0474047
PI3K/AKT Signaling Panel	Nanostring Technologies, Inc	GMX-PROMOD-NCTHPI3K-12, Lot# 0474053
COVID-19 GeoMx-formatted Antibody Panel including (TMPRSS2, clone EPR3861; ACE2, clone EPR4436; Cathepsin L/V/K/H, clone EPR8011; DDX5, clone EPR7239; and SARS-CoV-2 spike glycoprotein, polyclonal)	Abcam	ab273594, Lot# GR3347471-1
GeoMx Solid Tumor TME Morphology Kit	Nanostring Technologies, Inc	GMX-PRO-MORPH-HST-12; Item 121300310
Alexa Fluor® 647 alpha-Smooth Muscle Actin Antibody, clone 1A4	Novus Bio	IC1420R
**Biological samples**		
Autopsy tissues	Weill Cornell Medicine Department of Pathology	https://pathology.weill.cornell.edu/
**Chemicals, peptides, and recombinant proteins**		
TRIzol	Invitrogen	Cat. #15596026
10% neutral buffered Formalin	Electron Microscopy Sciences	Cat. #15712
DNAse I	Zymo Research	Cat. #E1010
**Critical commercial assays**		
Super-Script III Platinum SYBR Green One-Step qRT-PCR Kit	Invitrogen	Cat. #12594025
BD Univeral Viral Transport Media System	Becton, Dickinson and Company	Cat. #220526
QIAsymphony DSP Virus/Pathogen Mini Kit	Qiagen	Cat. #937036
NEBNext® rRNA Depletion Kit v2 (Human/Mouse/Rat) with RNA Sample Purification Beads	New England BioLabs	Cat. #E7405
NEBNext® Ultra^{TM} II Directional RNA Library Prep Kit for Illumina	New England BioLabs	Cat. #E7760
TapeStation 2200	Agilent Technologies	Cat. #G2964AA
Kapa Biosystems Illumina library quantification kit	Roche	Cat. 07960140001
GeoMx DSP system	Nanostring Technologies, Inc	MAN-10088-03
**Deposited data**		
Raw and analyzed RNA-seq data	This paper	dbGAP: accession #38851 and ID phs002258.v1.p1

(Continued on next page)

Continued

REAGENT or RESOURCE	SOURCE	IDENTIFIER
Analyzed Nanostring GeoMx data	This paper	GEO: GSE169504
Human reference genome NCBI build 38, Gencode Human Release 33 (GRCH38.p13)	Genome Reference Consortium	http://www.ncbi.nlm.nih.gov/projects/genome/assembly/grc/human/
Raw RNA-seq data	Rother et al.[44]	GSE159678
Reference scRNA-seq data	Travaglini et al.[45]; MacParland et al.[46]; Stewart et al.[47]; Wang et al.[8]	https://www.humancellatlas.org/
Molecular Signatures for GSEA (MSigDB)	Liberzon et al.[48]; Subramanian et al.[49]; Kuleshov et al.[50]; Sergushichev et al., 2016	http://www.gsea-msigdb.org/gsea/
Oligonucleotides		
Primers for RT-PCR; ACTB-Forward: CGTCACCAACTGGGACGACA	This paper	N/A
Primers for RT-PCR; ACTB-Reverse: CTTCTCGCGGTTGGCCTTGG	This paper	N/A
Primers for RT-PCR; *SARS-CoV-2-TRS-L*: CTCTTGTAGATCTGTTCTCTAAACGAAC	This paper	N/A
Primers for RT-PCR; *SARS-CoV-2-TRS-N*: GGTCCACCAAACGTAATGCG	This paper	N/A
Software and algorithms		
ImageJ	Schneider et al., 2012	https://imagej.nih.gov/ij/
nf-core/rnaseq pipeline	Ewels et al.[51]	https://nf-co.re/rnaseq
FastQC	Andrews[52]	https://www.bioinformatics.babraham.ac.uk/projects/fastqc/
Trim Galore!	N/A	https://github.com/FelixKrueger/TrimGalore
STAR	Dobin et al.[53]	https://github.com/alexdobin/STAR
Salmon	Patro et al.[54]	https://salmon.readthedocs.io/en/latest/salmon.html
Picard	N/A	https://github.com/broadinstitute/picard
StringTie	Kovaka et al.[55]	https://ccb.jhu.edu/software/stringtie/
Samtools	Li and Durbin[56]	http://samtools.sourceforge.net/
DESeq2 R package	Love et al.[57]	https://bioconductor.org/packages/release/bioc/html/DESeq2.html
MuSiC R package	Wang et al.[58]	https://xuranw.github.io/MuSiC/articles/MuSiC.html
quanTIseq R package	Finotello et al.[59]	https://icbi.i-med.ac.at/software/quantiseq/doc/
Cocor R package	Diedenhofen and Musch[60]	https://cran.r-project.org/web/packages/cocor/cocor.pdf
synRNASeqNet R package	Luciano Garofano	https://github.com/cran/synRNASeqNet
Other		
Resource page to visualize and explore autopsy RNA-seq data	This paper	https://covidgenes.weill.cornell.edu

RESOURCE AVAILABILITY

Lead contact

Further information and requests for resources and reagents should be directed to the lead contact, Christopher E. Mason (chm2042@med.cornell.edu).

Materials availability

This study does not involve new unique reagents or materials.

Data and code availability

All the raw sequence files and metadata for specimens, including per-run metrics and QC data, have been submitted to the database of Genotypes and Phenotypes dbGAP (accession #38851 and ID phs002258.v1.p1): https://www.ncbi.nlm.nih.gov/projects/gap/cgi-bin/study.cgi?study_id=phs002258.v1.p1. Nanostring GeoMx data are also deposited in the GEO database (GSE169504). Processed bulk RNA-seq data is also available online for simple visualization and exploration of gene expression and enriched pathways (https://covidgenes.weill.cornell.edu/). This is also available from Mendeley Data: https://dx.doi.org/10.17632/f4wh42nshy.2. Any additional information required to reanalyze the data reported in this work is available from the Lead Contact upon request.

EXPERIMENTAL MODEL AND SUBJECT DETAILS

IRB statement

Tissue samples were provided by the Weill Cornell Medicine Department of Pathology. The Tissue Procurement Facility operates under Institutional Review Board (IRB) approved protocol and follows guidelines set by Health Insurance Portability and Accountability Act (HIPAA). Experiments using samples from human subjects were conducted in accordance with local regulations and with the approval of the IRB at the Weill Cornell Medicine. The autopsy samples are considered human tissue research and were collected under IRB protocols 20-04021814 and 19-11021069. All autopsies have consent for research use from next of kin, and these studies were determined as exempt by IRB at Weill Cornell Medicine under those protocol numbers.

Patient sample collection

All autopsies are performed with consent of next of kin and permission for retention and research use of tissue. Autopsies were performed in a negative pressure room with protective equipment including N-95 masks; brain and bone were not obtained for safety reasons. All fresh tissues were procured prior to fixation and directly into Trizol for downstream RNA extraction. Tissues were collected from lung, liver, lymph nodes, kidney, and the heart as consent permitted. For GeoMx, RNAscope, trichrome and histology tissue sections were fixed in 10% neutral buffered formalin for 48 hours before processing and sectioning. These cases had a post-mortem interval of less than 48 hours. For bulk RNA-seq tissues, post-mortem intervals ranged from less than 24 hours to 72 hours (with 2 exceptions - one at 4 and one at 7 days - but passing RNA quality metrics) with an average of 2.5 days. All deceased patient remains were refrigerated at 4°C prior to autopsy performance.

METHOD DETAILS

qRT-PCR

Total RNA was extracted in TRIzol (Invitrogen) according to the manufacturer's instructions. To quantify viral replication, measured by the expression of sgRNA transcription of the viral N gene, one-step quantitative real-time PCR was performed using SuperScript III Platinum SYBR Green One-Step qRT-PCR Kit (Invitrogen) with primers specific for the TRS-L and TRS-B sites for the N gene as well as ACTB as an internal reference. Quantitative real-time PCR reactions were performed on an Applied Biosystems QuantStudio 6 Flex Real-Time PCR Instrument (ABI). Delta-delta-cycle threshold (ΔΔCT) was determined relative to ACTB levels and normalized to mock infected samples. Error bars indicate the standard deviation of the mean from three biological replicates. The sequences of primers/probes are provided in the key resources table.

RNA-seq analysis

Patient specimens were processed as described in Butler et al.[4] Clinical metadata is summarized in Table S1. Briefly, nasopharyngeal (NP) swabs were collected N using the BD Universal Viral Transport Media system (Becton, Dickinson and Company, Franklin Lakes, NJ) from symptomatic patients. Total Nucleic Acid (TNA) was extracted from using automated nucleic acid extraction on the QIAsymphony and the DSP Virus/Pathogen Mini Kit (Qiagen). Autopsy tissues were collected from lung, liver, lymph nodes, kidney, and the heart and were placed directly into Trizol, homogenized, and then snap frozen in liquid nitrogen. At least after 24 hours these tissue samples were then processed via standard protocols to isolate RNA.

For RNA library preparation, all samples' TNA were treated with DNAse 1 (Zymo Research, Catalog #E1010). Post-DNAse digested samples were then put into the NEBNext rRNA depletion v2 (Human/Mouse/Rat), Ultra II Directional RNA (10 ng), and Unique Dual Index Primer Pairs were used following the vendor protocols from New England Biolabs. Completed libraries were quantified by Qubit and run on a Bioanalyzer for size determination. Libraries were pooled and sent to the WCM Genomics Core or HudsonAlpha for final quantification by Qubit fluorometer (ThermoFisher Scientific), TapeStation 2200 (Agilent), and qRT-PCR using the Kapa Biosystems Illumina library quantification kit.

NYGC RNA sequencing libraries were prepared using the KAPA Hyper Library Preparation Kit + RiboErase, HMR (Roche) in accordance with manufacturer's recommendations. Briefly, 50-200ng of Total RNA were used for ribosomal depletion and fragmentation. Depleted RNA underwent first and second strand cDNA synthesis followed by adenylation, and ligation of unique dual indexed adapters. Libraries were amplified using 12 cycles of PCR and cleaned-up by magnetic bead purification. Final libraries were quantified using fluorescent-based assays including PicoGreen (Life Technologies) or Qubit Fluorometer (invitrogen) and Fragment Analyzer (Advanced Analytics) and sequenced on a NovaSeq 6000 sequencer (v1 chemistry) with 2x150bp targeting 60M reads per sample.

Spatial transcriptomics analysis

Gene Expression profiling of freshly extracted RNA from formalin fixed paraffin-embedded (FFPE) lung samples was performed using the NanoString PanCancer IO360 panel with custom probes for SARS-CoV-2 viral genes. After normalization, high and low COVID-19 clusters were identified by unsupervised analysis, and samples from each cluster were selected for additional profiling. GeoMx Digital Spatial Profiling (DSP) was performed on these samples, and control samples from non-viral ARDS, influenza, and normal lung tissues following standard protocols using the COVID-19 Immune Response Atlas.[61,62] Samples were stained with immunofluorescent antibodies for CD68, CD45, PanCK, and DNA (Syto-13). Regions profiled included vascular zone, large airway, alveoli zone, and IF-guided segments focused specifically on macrophages. Samples were sequenced on an Illumina NextSeq, processed and filtered for quality as described in supplementary methods. Differential expression was assessed on the resulting normalized data using mixed effect models, accounting for intra-patient heterogeneity to assess differences between SARS-CoV-2 high and low viral load samples, and among distinct tissue structures profiled. Cell deconvolution of the GeoMx data was performed using the SpatialDecon R package.[63] Gene set enrichment analysis (GSEA) was performed to qualify coordinate gene expression changes quantified during differential expression analysis.[64]

QUANTIFICATION AND STATISTICAL ANALYSIS

RNA-seq analysis

Differential gene analysis

RNAseq data was processed through the nf-core/rnaseq pipeline.[51] This workflow involved quality control of the reads with FastQC,[52] adapter trimming using Trim Galore (https://github.com/FelixKrueger/TrimGalore), read alignment with STAR,[53] gene quantification with Salmon,[54] duplicate read marking with Picard MarkDuplicates (https://github.com/broadinstitute/picard), and transcript quantification with StringTie.[55] Other quality control measures included RSeQC, Qualimap, and dupRadar. Alignment was performed using the GRCh38 build native to nf-core and annotation was performed using Gencode Human Release 33 (GRCH38.p13). FeatureCounts reads were normalized using variance-stabilizing transform (vst) in DESeq2 package in R for visualization purposes in log-scale.[57] Cell deconvolution was performed using MuSiC on single cell reference datasets for lung, liver, kidney, and heart.[8,45–47,58] Immune cell deconvolution was performed on lymph node samples using quanTIseq.[59] Differential expression of genes was calculated by DESeq2. Differential expression comparisons were done as either COVID + cases versus COVID- controls for each tissue specifically, correcting for sequencing batches with a covariate where applicable, or pairwise comparison of viral levels from the lung as determined by nCounter data. In the volcano plot protein coding genes were plotted using Gencode classifications using -log10 (adjusted value) and log2 fold-change metrics. Genes with BH-adjusted p value < 0.01 and absolute log2 fold-change greater than 0.58 (at least 50% change in either direction) were taken as significantly differentially regulated.[65] Genes were ranked by their Wald statistic and their log2 fold-change values and used as input for gene set enrichment analysis (GSEA) on the molecular signatures database (MSigDB).[64,48–50] Any signature with adjusted p value < 0.01 was taken as significant. List of differentially expressed genes and significantly enriched pathways are reported in Table S2.

Pairwise correlations of cell types by conditions

Correlation matrix visualizes the Pearson correlation coefficient by cell types within each disease condition. Statistically insignificant correlations (p-value > 0.05) are filtered and identified clusters of positive and negative correlation is marked. The correlations from SARS-CoV-2 high and low viral load samples are compared with normal, using R package cocor (v1.1-3).[60] Briefly, the correlation coefficients are tested using Fisher's r-to-Z transformation to quantify the differences between the two correlations. To quantify correlations, each data point (or correlation coefficient) corresponds to a fisher-tested correlation (z statistics and –log(P-value) for x and y axes, respectively). The entropy calculations were done with the synRNASeqNet R package (v1.0, entropyML function, https://github.com/cran/synRNASeqNet). The deconvoluted cell counts were used as an input to run maximum likelihood entropy calculations.

Similarity analysis

The consensus gene profiles for alveolar, large airway, and vascular healthy samples were built by taking the average gene profiles from healthy ROIs of respective tissue origin. To validate gene profiles, healthy ROIs were randomly sampled (1, 5, and 10 ROIs) and compared with the consensus profiles. With these gene profiles, we assessed the similarity of the profile from each ROI with the reference profile by taking cosine similarity (1 being closer to the reference, 0 being orthogonal, Figure S5A). To identify genes specific to tissue- and disease- states, we performed logistic regression with L1 norm to model the gene expression profiles. The logistic regression was done with glmnet (v4.1-1).[66] The genes with highest coefficients were filtered to identify 35 genes that may distinguish diseased tissue types (Table S3).

GeoMx transcriptomic data normalization and quantification

Spatial transcriptomics analysis

Discerning viral load from bulk nCounter screening—Bulk expression profiling was performed to identify COVID-19 patients with high vs low viral load. To do this, RNA from fresh TRIzol extracted and fixed lung tissue from 29 COVID-19 autopsies, 4 non-COVID-19 lung injury and 3 controls were evaluated by bulk expression analysis using NanoString's nCounter PanCancer IO360 Panel plus custom probes for eight SARS-CoV-2 viral genes (encoding ORF7a, surface glycoprotein, nucleocapsid phosphoprotein, ORF8, envelope

protein, ORF3a, membrane glycoprotein, and ORF1ab) to assess viral content. At least 100 ng of RNA was loaded for hybridization and quantified by the nCounter MAX Analysis System (NanoString Technologies, Seattle, WA, USA).

Transcript counts were normalized to ERCC positive controls and housekeeper reference gene expression prior to analysis. Hierarchical clustering of nCounter results revealed two clusters of high and low severity and four "SARS-CoV-2 high" and four "SARS-CoV-2 low" patients were selected (Figure S1D). These eight samples were analyzed with two influenza-infected patients, three non-viral ARDS patients, and three normal lung control patients using the GeoMx platform.

RNA/NGS slide preparation for GeoMx DSP

For GeoMx DSP slide preparation, we followed the GeoMx DSP slide prep user manual (MAN-10087-04). Briefly, tissue slides were baked in a drying oven at 60 °C for 1 hour and then loaded to Leica Biosystems BOND RX FFPE for deparaffinization and rehydration. After the target retrieval step, tissues were treated with Proteinase K solution to expose RNA targets followed by fixation with 10% NBF. After all tissue pre-treatments were done, tissue slides were unloaded from the Leica Biosystems BOND RX and incubated with RNA probe mix (COVID-19 Immune Response Atlas panel) overnight. The next day, tissues were washed and stained with tissue visualization markers; CD68-647 at 1:400 (Novus Bio, NBP2-34736AF647), CD45-594 at 1:10 (NanoString Technologies), PanCK-532 at 1:20 (NanoString Technologies) and/or SYTO 13 at 1:10 (Thermo Scientific S7575).

GeoMx DSP sample collections

For GeoMx DSP sample collections, we followed the GeoMx DSP instrument user manual (MAN-10088-03). Briefly, tissue slides were loaded on the GeoMx DSP instrument and then scanned to visualize whole tissue images. For each tissue sample, we collected 4 types of functional tissue regions: vascular zone, large airway, alveoli zone, and macrophages. Each tissue region was carefully selected by a board-certified pathologist. Regions of interest (ROIs) were then segmented with corresponding fluorescent tissue markers, when available within the region. Twenty-four to twenty-three GeoMx DSP regions were selected per tissue and collected following UV illumination within the defined segment as described in Merritt et al.[62] Compartments that were segmented within a region of interest were collected separately as unique areas of illumination (AOIs).

GeoMx DSP NGS library preparation and sequencing

Each GeoMx DSP sample was uniquely indexed using Illumina's i5 x i7 dual-indexing system. 4 μL of a GeoMx DSP sample was used in a PCR reaction with 1 μM of i5 primer, 1 μM i7 primer, and 1× NSTG PCR Master Mix. Thermocycler conditions were 37 °C for 30 min, 50 °C for 10 min, 95 °C for 3 min, 18 cycles of 95 °C for 15 sec, 65 °C for 60 sec, 68 °C for 30 sec, and final extension of 68 °C for 5 min. PCR reactions were purified with two rounds of AMPure XP beads (Beckman Coulter) at 1.2× bead-to-sample ratio. Libraries were paired end sequenced (2 × 75) on a NextSeq550 up to 400 million total aligned reads.

Processing and filtering raw NGS data

Three hundred seventy-nine AOIs plus non-template controls (NTCs) were sequenced, producing about 1.3B reads (with about ~11% unique). NextSeq-derived FASTQ files for each sample were compiled for each AOI using Illumina's bcl2fastq program and then demultiplexed and converted to Digital Count Conversion (DCC) files using Nanostring's GeoMx DnD pipeline (v1). These DCC files were then converted to an expression count matrix using a custom python script. A minimum of 10,000 reads were required for each non-NTC sample (2 AOIs removed). Probes were checked for outlier status by implementing a global Grubb's outlier test with alpha set to 0.01. The counts for all remaining probes for a given target were then collapsed into a single metric by taking the geometric mean of probe counts. A count of 1 was added to any probe that yielded 0 counts before the geometric mean was taken. For each sample, RNA probe pool specific negative probe normalization factor was generated based on the geometric mean of negative probes in each pool.

Quality control and AOI filtering

Following initial screening above, there were 373 AOIs interrogated using DSP that span 16 patients and three compartments (288 alveolar, 48 large airway, and 37 vascular regions). Of these, 370 AOIs yielded greater than 50 nuclei. The 75th percentile of the gene counts (i.e., geometric mean across all non-outlier probes for a given gene) for each AOI were calculated and normalized to the geometric mean of the 75th percentile across all AOIs to give the upper quartile or Q3 normalization factors for each AOI. The distribution of these Q3 normalization factors were then checked for outliers defined as any AOI greater than two standard deviations from the mean log2 Q3 normalization factor. This criterion removed 15 AOIs that fell below the range and 1 AOI that fell above the range. Following AOI filtering, 358 (of 373, ~96%) AOIs were used for downstream analyses.

Removal of gene outliers and normalization

Gene outliers were detected by a limit of quantification (LOQ) approach. The LOQ for each AOI was defined as the sample's negative geometric mean multiplied by its negative standard deviation raised to the power of two. Any target (1,837 total) that was not above LOQ in at least 2% of AOIs were deemed prohibitively low expressors and were removed from analysis. This feature-based filtering approach discarded 171 genes (9.3%) leaving 1,666 genes. Genes were normalized by the Q3 approach as above.

Deconvolution of cell proportions using GeoMx

Cell deconvolution methods followed that of Desai et al.[15] Cell mixing proportions were performed using the R package SpatialDecon[63] using the cell profile matrix based upon the Human Cell Atlas adult lung 10× dataset and appended with a neutrophil profile derived from snRNA-seq of lung tumors.[67] ROIs were selected to be representative across the FFPE slides, based on morphology and immunofluorescence of each tissue.

Differential expression analysis

Two different sets of differential expression (DE) analyses were performed. Common in both sets of models were five groups (COVID-19 SARS-CoV-2 high, SARS-CoV-2 low, influenza, Non-viral ARDS, and Normal) and three compartments (alveolar, large airway, vascular). In DE set one, differences between all 10 pairwise groupings were performed to look for differences between pairwise comparisons and to serve as the basis for downstream gene set enrichment analysis (GSEA). In the second DE set, SARS-CoV-2 high and low viral load groups were compared against one of three non-COVID groupings to identify genes that are up- or downregulated in COVID-19 (*sensu lato*) relative to non-COVID-19 groups and to identify genes that are consistently differentially expressed in SARS-CoV-2 high and in low separately.

In the first model, each 10 pairwise groupings were considered separately (e.g., SARS-CoV-2 low vs SARS-CoV-2 high). DE analysis was performed by fitting each gene's normalized log2 expression level using a Linear Mixed Effect model to account for interpatient variation with the R package lmerTest.[6] Patient ID was used as a random effect (random intercept) and grouping, compartment, and grouping-by-compartment interactions were used as fixed effects. Satterthwaite's approximation for degrees of freedom for P-value calculation was used.[68]

In the second set of models, SARS-CoV-2 high and SARS-CoV-2 low AOIs were always included, and DE was used to determine how genes were up- or downregulated compared to each of three non-COVID groups. As such, there were three different "sets" (influenza vs SARS-CoV-2 high vs SARS-CoV-2 low; Non-viral ARDS vs SARS-CoV-2 high vs SARS-CoV-2 low; and Normal vs SARS-CoV-2 high vs SARS-CoV-2 low). For a given gene and a given set, AOIs were first filtered to exclude non-members (i.e., exclude normal and Non-viral ARDS in the set "influenza vs SARS-CoV-2 high vs SARS-CoV-2 low"). The log2 expression of sample for a given gene was fit to a mixed effect model with group (three levels) and compartment (three levels) and their interaction as fixed effects and Patient ID as a random effect. The Least Squares (LS) means or "marginal means" were estimated for each comparison (i.e., log2 means for levels of "group" which are averaged over the levels of other factors in the model[68]). In addition to the LS means, the pairwise P-values for all 3 comparisons were computed.

To visualize the marginal means for each gene relative to SARS-CoV-2 high, SARS-CoV-2 low, and a given normal group, the three-dimensional data were collapsed into two-dimensional ternary plots. Specifically, for a given gene g in set S, the three-element vector of marginal means can be expressed as g_S. By convention, the order of the elements of g_S were normal, SARS-CoV-2 high, and SARS-CoV-2 low. Elements of g_S were then rescaled while preserving their relative relationship by multiplying by a scaling factor and converting from log2 space to linear space. Then g_S was scaled further by dividing each element by the element with the minimum value. To convert g_S from a vector of three to a vector of two (representing points along a simplex plane), the new x coordinates were calculated by:

$$x = \frac{g_{S_2} + \frac{1}{2} g_{S_3}}{\sum_{i=1}^{i=3} g_{S_i}}$$

where represents the ith element in vector g_S. Similarly, the y coordinates were calculated by:

$$y = \frac{g_{S_3}\sqrt{\frac{3}{4}}}{\sum_{i=1}^{i=3} g_{S_i}}$$

A given gene is then assigned a "corner" by how close it is from each of the three simplex's corners.

Each gene has three p-values associated with it (from the pairwise contrasts above). The contrast with the lowest p-value was selected to represent a given gene (i.e., this corresponded well with the corner that the gene was assigned to). P-values were then adjusted to account for multiple comparisons by using the Benjamini-Hochberg procedure.[65] Differentially expressed genes from this analysis is included in Table S3.

Gene set enrichment

MA plots[69] from the 10 pairwise DE analyses (DE model 1) were used to ensure that low expressors were not accounting for the majority of the large fold changes. Gene Set Enrichment Analysis (GSEA) was conducted using the R package fGSEA[64] with MSigDB Hallmark and Reactome[70] databases. Gene ranks were based on the log2 fold change from the individual DE analyses and gene sets were bound between 15 and 500 genes. P-values for enrichment were estimated by 1,000 permutations of the data. The pathways were then sorted based on adjusted p-value first and then by their Normalized Expression Score (NES).

For the 10 most extreme pathways in each direction as well as the COVID-19 spike-in genes, single sample GSEA (ssGSEA) was performed using the R package gsva[71] with a min and max size of 15 and 500, respectively. Enrichment scores for a given pathway were rescaled by dividing AOI's enrichment score by the mean across samples and then rescaled between 0 and 1. These rescaled enrichment scores were then visualized for each AOIs' x and y coordinates from their respective FFPE slide.

Histology and imaging analysis

Sections from SARS-CoV-2 high (4), SARS-CoV-2 low (4) and normal lungs (3) cases used for the GeoMx analysis were stained using hematoxylin and eosin and Masson'|'s Trichrome according to standard protocol. Four 20× regions were randomly selected from each slide the Color deconvolution2 algorithm for ImageJ[72] and cellular trichrome rich areas were manually selected and measured for pixel area (cellular areas on red deconvolution, trichome on blue deconvolution). Total image pixel area was used to determine percent fibroblast-rich trichrome positive zones.

Correlation plot comparing different COVID-19 samples and tissues

To generate the correlation plot comparing the global changes for COVID-19 infection between different tissues, we utilized several different RNA-sequence data. The collection of the RNA sequencing data for the NP swab samples were described previously.[4] The NP swab samples were analyzed with different methodologies with one comparing COVID-19 viral infection to the negative patients and the other a regression on continuous variables as a function of SARS-CoV-2 sequence amount. The viral comparison was previously described in Butler et al.[4] and the DESeq2[57] was utilized to generate the differential expression data.

The monocyte COVID-19 RNA-Seq data, published under the accession GSE159678,[44] was downloaded from SRA and gene expression was quantified using Salmon's selective alignment approach.[54] The RNA-Seq processing pipeline was implemented using pyrpipe (https://github.com/urmi-21/pyrpipe/tree/master/case_studies/Covid_RNA-Seq).[73] Exploratory data analysis and differential expression analysis were performed using MetaOmGraph.[74] From the differential expression analysis for each group the fold-change values for the genes were filtered with an adjusted p-value < 0.05. A correlation plot between the fold-change values for the significantly regulated genes for each comparison of the COVID-19 samples were plotted using R program corrplot v0.84.

Viral genome analysis

Total RNA-seq reads were classified against a custom, pan-kingdom reference using kraken2.[75] Reads that mapped uniquely to SARS-CoV-2 were aligned to the Wuhan reference genome using bwa-mem.[56] Alignments were deduplicated, assembled, and called for major variants (Variant Allele Frequency > 0.6) using IVAR[76] with a minimum coverage of 5 reads per site.

Cell Reports Medicine, Volume *3*

Supplemental information

System-wide transcriptome damage and tissue identity loss in COVID-19 patients

Jiwoon Park, Jonathan Foox, Tyler Hether, David C. Danko, Sarah Warren, Youngmi Kim, Jason Reeves, Daniel J. Butler, Christopher Mozsary, Joel Rosiene, Alon Shaiber, Evan E. Afshin, Matthew MacKay, André F. Rendeiro, Yaron Bram, Vasuretha Chandar, Heather Geiger, Arryn Craney, Priya Velu, Ari M. Melnick, Iman Hajirasouliha, Afshin Beheshti, Deanne Taylor, Amanda Saravia-Butler, Urminder Singh, Eve Syrkin Wurtele, Jonathan Schisler, Samantha Fennessey, André Corvelo, Michael C. Zody, Soren Germer, Steven Salvatore, Shawn Levy, Shixiu Wu, Nicholas P. Tatonetti, Sagi Shapira, Mirella Salvatore, Lars F. Westblade, Melissa Cushing, Hanna Rennert, Alison J. Kriegel, Olivier Elemento, Marcin Imielinski, Charles M. Rice, Alain C. Borczuk, Cem Meydan, Robert E. Schwartz, and Christopher E. Mason

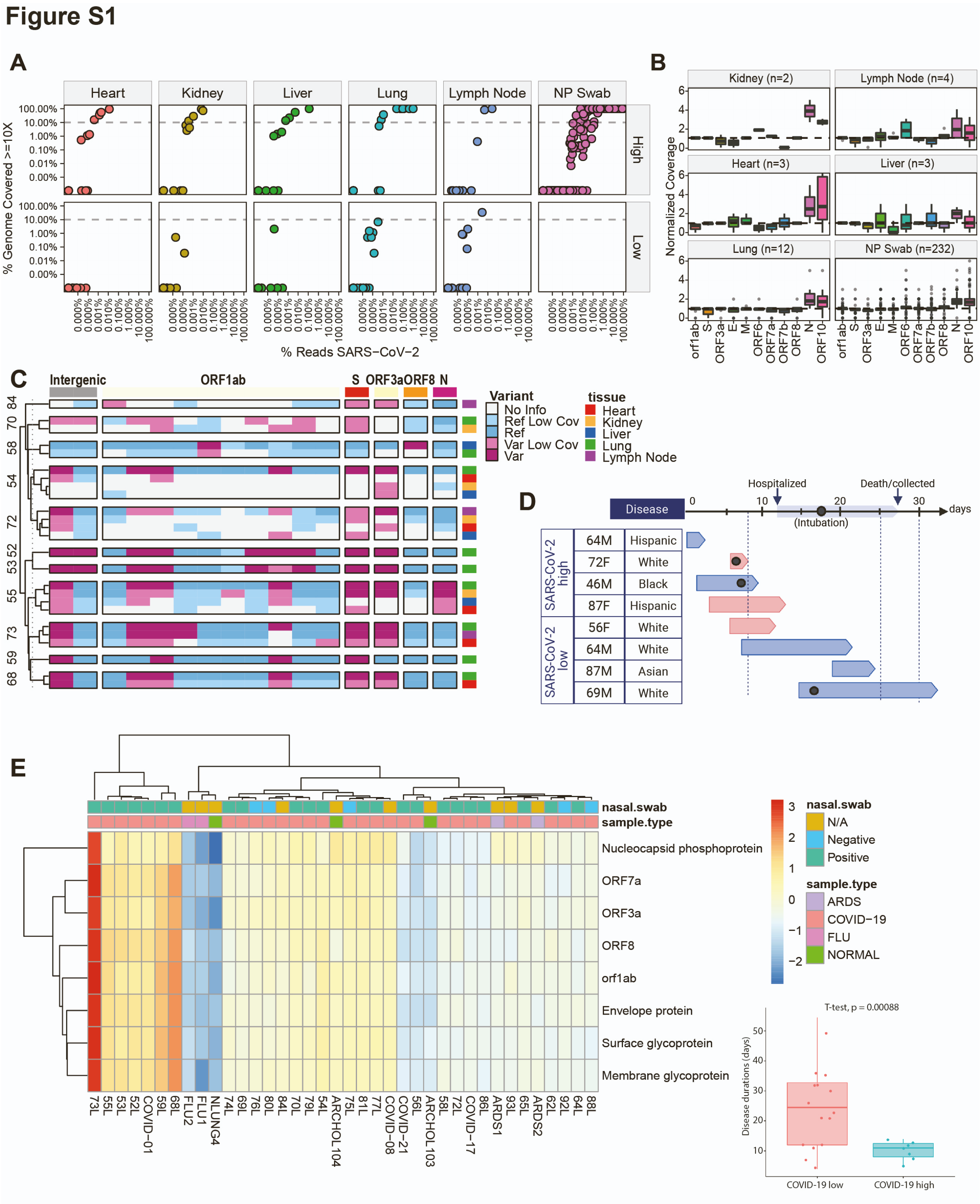

Figure S1, related to Figure 1. **Viral Genome Reconstruction from Total RNA-Seq Data and clinical course of the COVID-19 lung samples.** (**A**) For each patient sample per tissue (41 heart, 27 kidney, 40 liver, 40 lung, 27 lymph node tissues total), the percentage of Total RNA-seq reads mapped uniquely to SARS-CoV-2 and no other taxon is shown, against the percentage of the viral genome (Wuhan reference) that was covered with >10 reads during genome assembly. (**B**) Normalized expression value per gene body in the SARS-CoV-2 genome for samples where at least 10% of the viral genome was covered with >10 reads. (**C**) Heatmap of variant alleles across the viral genome, grouped by patient. (**D**) Summarized clinical course of the COVID-19 patients including four representative samples from high and low viral RNA load, which were used for GeoMx analysis. (**E**) ERCC and HK normalized nCounter gene expression values were centered and rescaled. The resulting Z-scores are shown in this unsupervised clustering. COVID-19 positivity, where applicable, is indicated above

columns. Bottom right included the summary of days of disease durations of each high/low groups (total n = 38, p-value obtained from paired t-test).

Figure S2

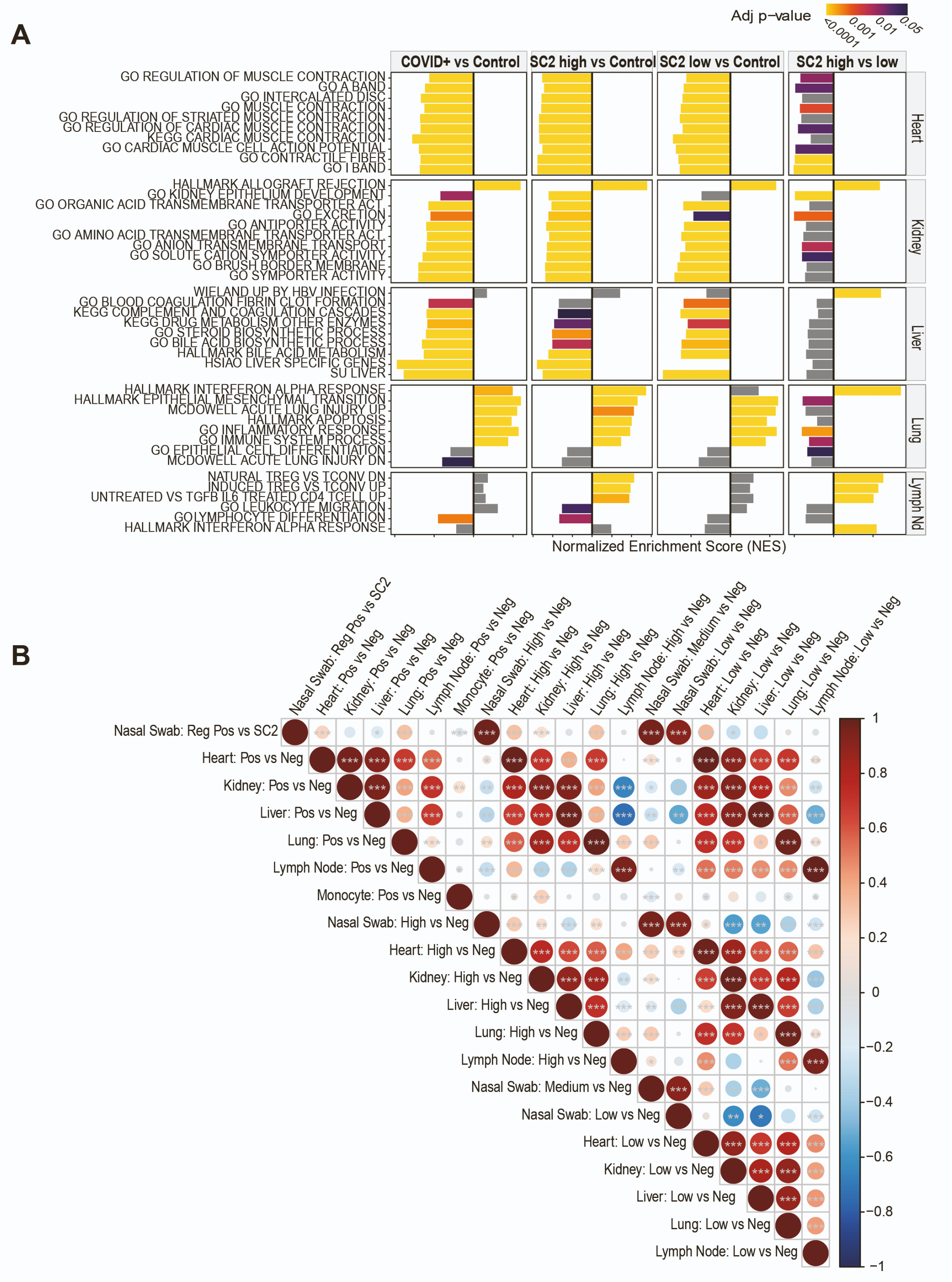

Figure S2, related to Figure 2. **Enriched pathways and correlations observed in COVID-19 by organ types and viral load comparisons**. (A) Select pathways that show significant differences in different tissues (41 heart, 27 kidney, 40 liver, 40 lung, 27 lymph node tissues total) are shown, with the statistical significance shown as a color range (legend, GSEA permutation test and adaptive multilevel splitting Monte Carlo method), and non-significant differences shown in grey. (B) Correlations between each sample type (label) gene expression matrix and other tissue/sample types are shown as a correlation range (red, high up to 1.0 and blue, low down to -1.0). The p-values represent the significance of the correlation with * p-value < 0.05, ** p-value < 0.01, and *** p-value < 0.001.

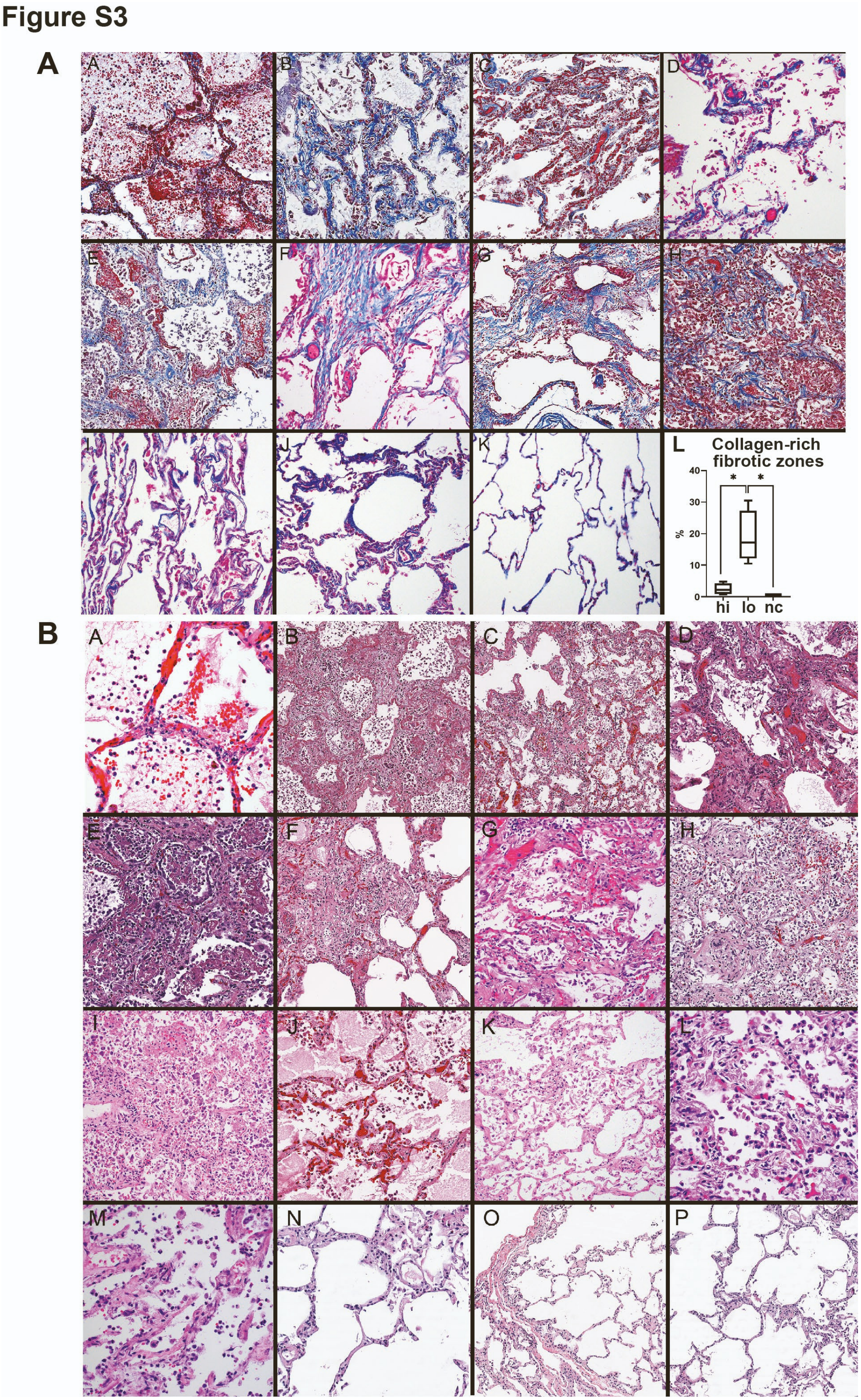

Figure S3, related to Figure 3. **Representative Trichrome stained lung tissue regions used for quantification and GeoMx spatial transcriptomics**. (A) Lung tissue regions for bulk quantification: A-D, COVID-19 with SARS-CoV-2 high cases: COVID1, COVID2, 55 and 59 showing mild increase in collagen zones with focally increased spindled cells, best seen in B in the figure. E-H, COVID-19 with SARS-CoV-2 low cases: COVID 17, 21, 73 and 86 with marked areas of increased

collagen containing spindled cells, best seen in F and G in the figure. I-K, normal lungs: 4, 103 and 104 showing blue staining collagen without expanded zones of cellularity (Masson trichrome, for all panels original magnification x 50). L, Quantification of collagen-rich fibroblastic zones, *:p-value < 0.01. (B) Representative lung morphology for GeoMx spatial profiling: A-H, COVID cases including COVID1, COVID2, 55L, 59L, COVID 17, COVID21, 73L, 86L; I-J, influenza induced ARDS: Flu1, Flu2; K-M, ARDS non-viral: ARDS1, ARDS2, ARDS3; N-P, Normal lung: NL4, Archoi103, Archoi 104. Hematoxylin and eosin stain, Original magnification x100 except for C and D in the figure (x50).

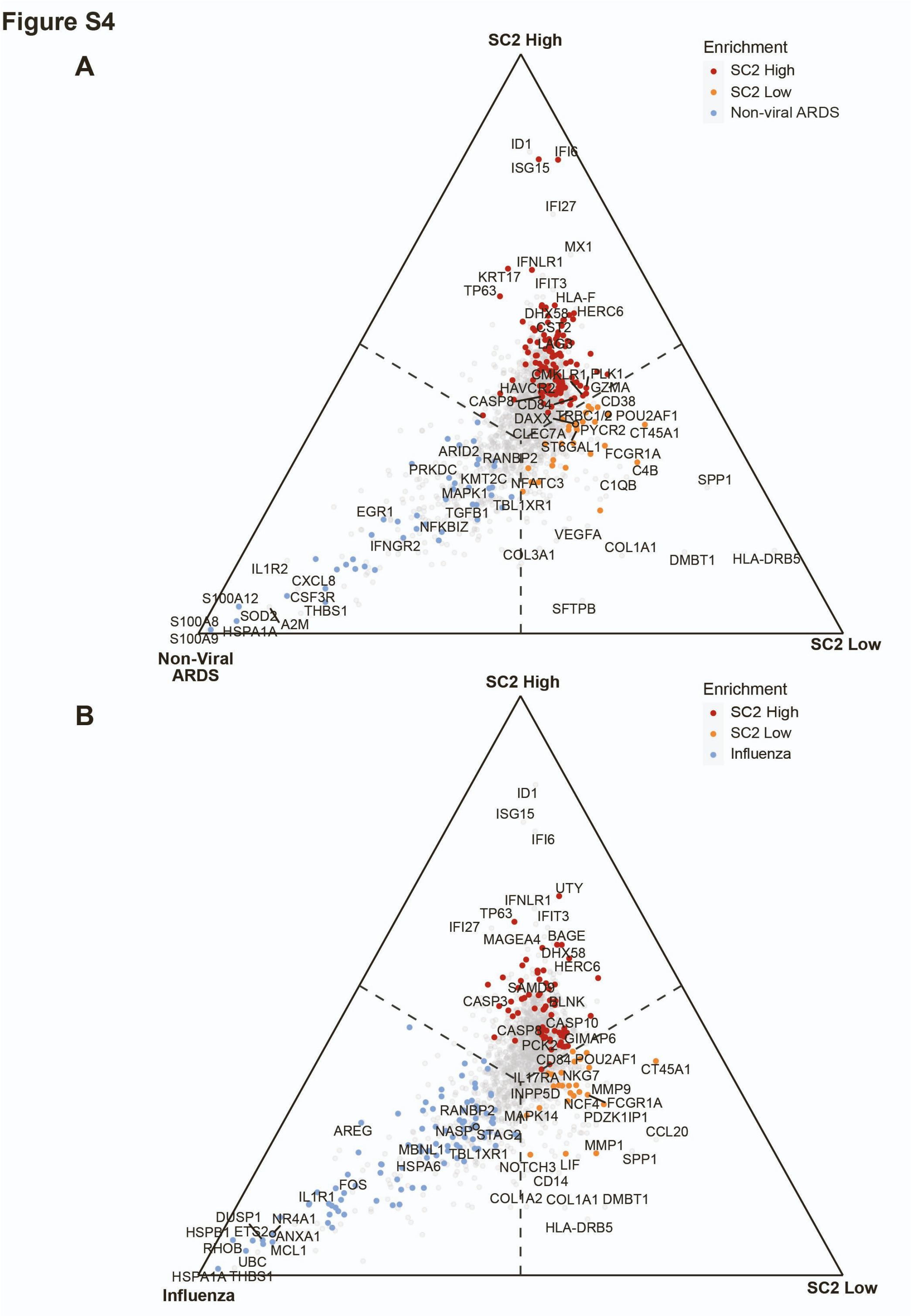

Figure S4, related to Figure 4. **Differentially expressed genes across three conditions**. (**A**) Ternary plot for influenza (n=2, 46 ROIs) vs COVID-19 SARS-CoV-2 high viral load (n=4, 86 ROIs) vs low viral load (n=4, 97 ROIs). (**B**) Ternary plot for non-viral ARDS (n=3, 67 ROIs) vs SARS-CoV-2 high (n=4, 86 ROIs) vs SARS-CoV-2 low (n=4, 97 ROIs).

Figure S5

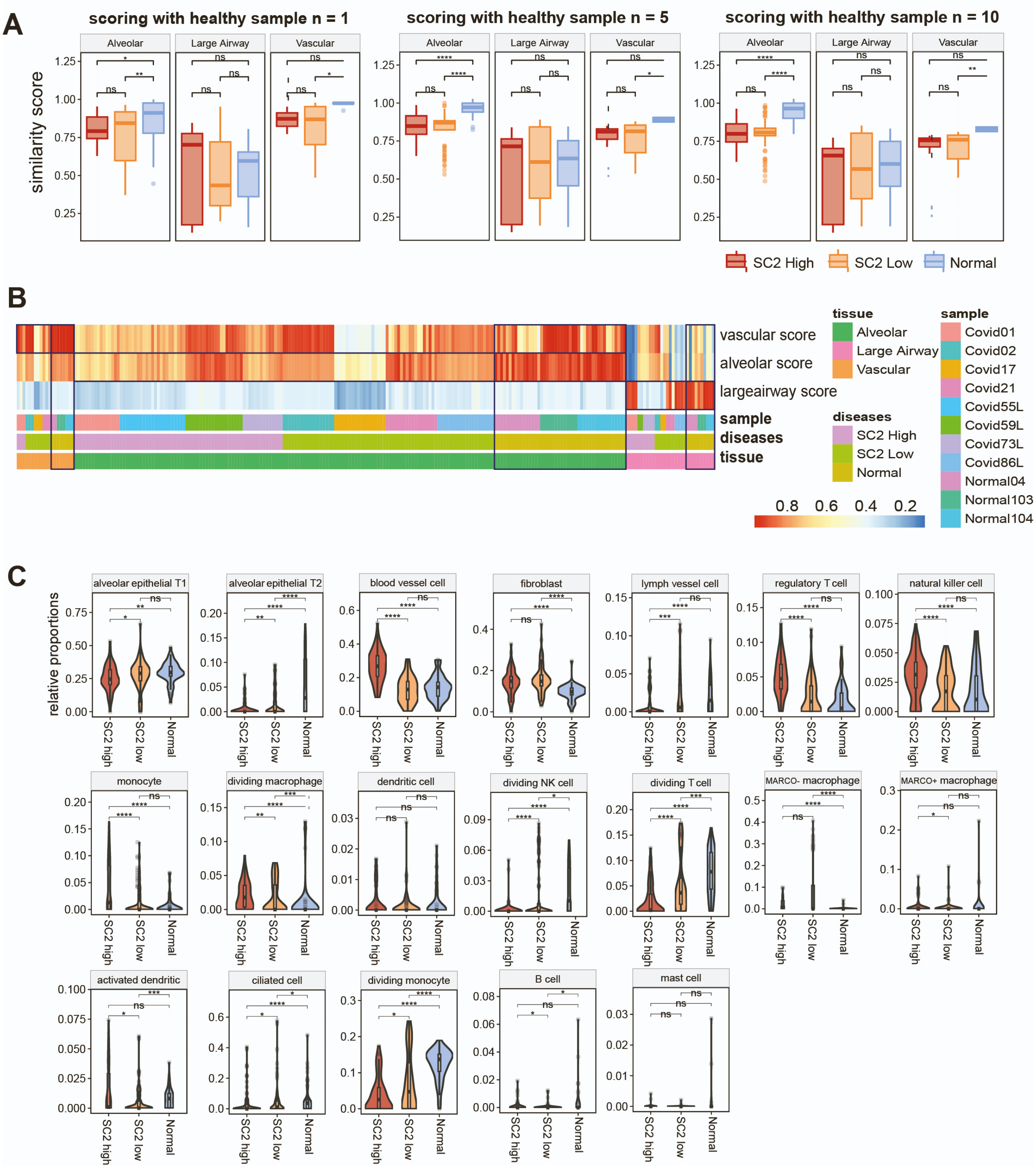

Figure S5, related to Figure 5. **Similarity score** visualizations and cell proportion changes from GeoMx. (**A**) Validation of reference healthy gene profile signature by randomly sampling healthy ROIs (64 ROIs total) for gene expression profiles. (**B**) Heatmap showing individual similarity scores across 64 ROIs. (**C**) Deconvoluted cell type proportions by conditions. From GeoMx data, cell proportions for each ROIs were plotted to compare between COVID-19 (183 ROIs) vs. normal control (64 ROIs). The median and quartiles are noted by the box plot inside. P-value two-tailed t-tests were done to compare the means (ns: non-significant, *:p <= 0.05, **: p <= 0.01, ***: p <= 0.001, and ****: p <= 0.0001).

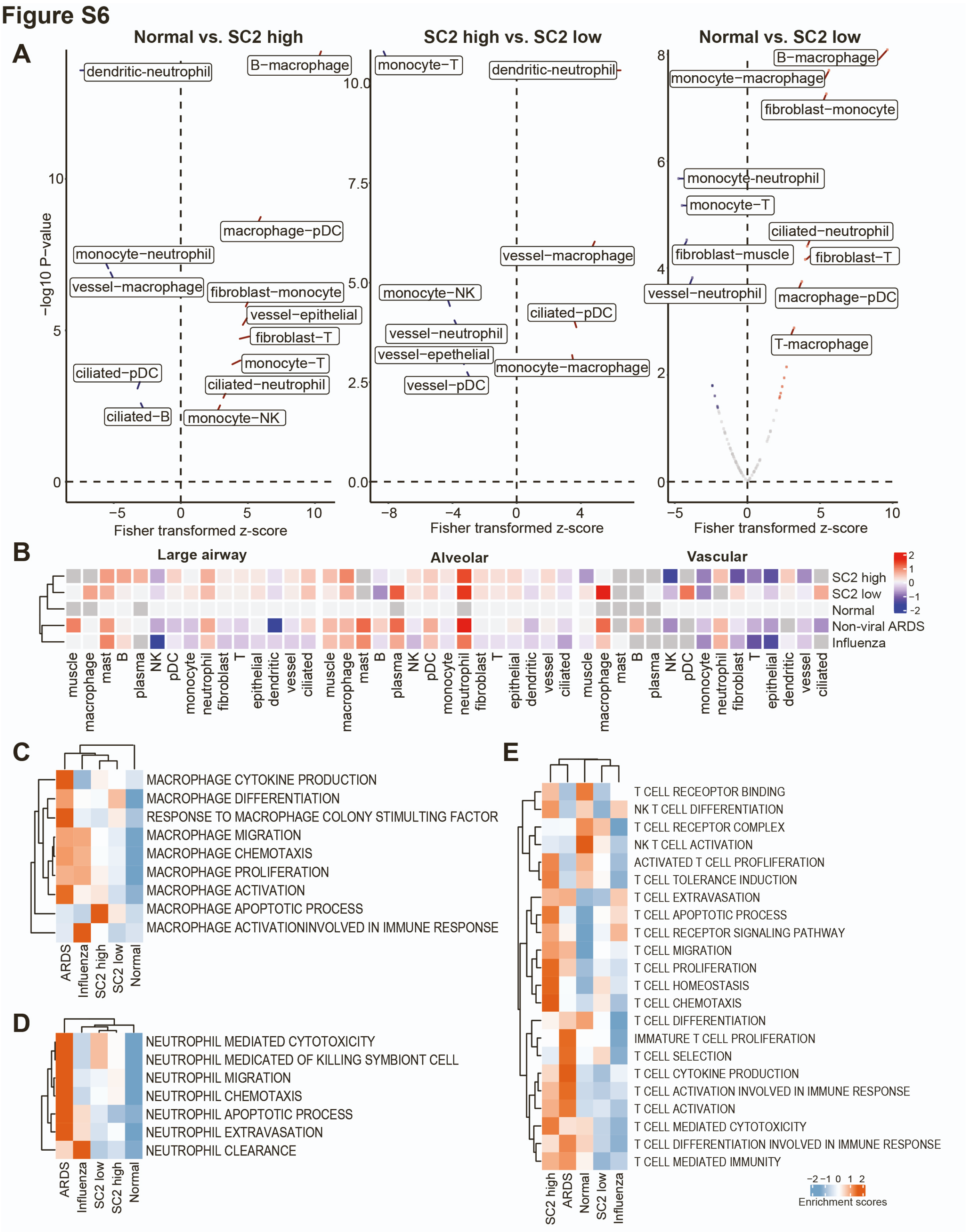

Figure S6, related to Figure 6. **Quantitative descriptions of correlations, cell-type counts entropy values, and ssGSEA scores by conditions**. (**A**) Quantitative, pairwise comparison of the cell type correlations. To quantitatively compare correlation patterns in Figure 6A, the correlations (from 64 normal ROIs, 86 and 97 COVID-19 high and low ROIs) are compared pairwise by Fisher's test. The transformed scores and p-values are shown. (**B**) Entropy Calculations (Maximum likelihood model) for cell-type counts of each condition: SARS-CoV-2 high (n=4, 86 ROIs), low (n=4, 97 ROIs), Influenza (n=2, 46 ROIs), and ARDS (n=3, 67 ROIs), relative to normal (n=3, 64 ROIs). The single-sample enrichment scores of (**C**) macrophage, (**D**) neutrophil, and (**E**) T and NK cell-related gene sets were evaluated. The scores were averaged and compared across SARS-CoV-2 high (n=4, 86 ROIs), low (n=4, 97 ROIs), Influenza (n=2, 46 ROIs), and ARDS (n=3, 67 ROIs), relative to normal (n=3, 64 ROIs).
